# Mucociliary Wnt signaling promotes cilia biogenesis and beating

**DOI:** 10.1038/s41467-023-36743-2

**Published:** 2023-03-06

**Authors:** Carina Seidl, Fabio Da Silva, Kaiqing Zhang, Kai Wohlgemuth, Heymut Omran, Christof Niehrs

**Affiliations:** 1 Division of Molecular Embryology, DKFZ-ZMBH Alliance, 69120 Heidelberg, Germany; 2 University Children’s Hospital Muenster, Department of General Pediatrics, 48149 Muenster, Germany; 3 Institute of Molecular Biology (IMB), 55128 Mainz, Germany

**Keywords:** Ciliogenesis, Cell signalling

## Abstract

It is widely thought that Wnt/Lrp6 signaling proceeds through the cytoplasm and that motile cilia are signaling-inert nanomotors. Contrasting both views, we here show in the mucociliary epidermis of *X. tropicalis* embryos that motile cilia transduce a ciliary Wnt signal that is distinct from canonical β-catenin signaling. Instead, it engages a Wnt-Gsk3-Ppp1r11-Pp1 signaling axis. Mucociliary Wnt signaling is essential for ciliogenesis and it engages Lrp6 co-receptors that localize to cilia via a VxP ciliary targeting sequence. Live-cell imaging using a ciliary Gsk3 biosensor reveals an immediate response of motile cilia to Wnt ligand. Wnt treatment stimulates ciliary beating in *X. tropicalis* embryos and primary human airway mucociliary epithelia. Moreover, Wnt treatment improves ciliary function in *X. tropicalis* ciliopathy models of male infertility and primary ciliary dyskinesia (*ccdc108*, *gas2l2)*. We conclude that *X. tropicalis* motile cilia are Wnt signaling organelles that transduce a distinct Wnt-Pp1 response.

## Abstract (web summary)

Motile cilia are thought of as signaling-inert nanomotors. Here, the authors show that motile cilia transduce a Wnt-protein phosphatase 1 signal that regulates cilia biogenesis and ciliary beating in *Xenopus* embryos and in human airway epithelia.

## Introduction

In processes ranging from larval development to respiration, mucociliary membranes defend epithelia against irritants and pathogens by a directional mucous flow generated through the coordinated beating of motile cilia^1,2^. Deficiency of these nanopropellers leads to airway disease and ciliopathies^3^, and understanding their cell biology is important for the rational design of cilio-stimulatory therapies^4^.

A highly specialized form of motile cilia are flagella of spermatozoa. *Cyclin Y-like 1* (*Ccnyl1*) mutant mice are male sterile^5,6^ and we elucidated that this is because Wnt signaling across the flagellar outer membrane is required to promote sperm maturation^5^. In addition, Wnt/β-catenin signaling promotes motile ciliogenesis in zebrafish, frog, and mouse via transcriptional gene activation of downstream regulators such as the transcription factor *Foxj1*^7–12^. However, the function of Wnt/Ccnyl1 in sperm flagellar morphogenesis is independent of β-catenin and transcription. Ccnyl1 and its close homolog Cyclin Y (Ccny) activate CDK14/16, which phosphorylate and prime the Wnt co-receptor LRP6 (low-density lipoprotein receptor-related protein 6) for incoming Wnt ligands mostly in β-catenin-independent Wnt/Gsk3 signaling^13,14^. This is also the case in spermatozoa, where Wnt/Gsk3 signaling orchestrates a rich post-transcriptional flagellar morphogenesis program^5^. Wnt signaling across the flagellar outer membrane is intriguing because unlike chemosensory primary cilia, motile cilia are with few exceptions^15,16^ considered as signaling-inert nanomotors. Flagellar Wnt signaling raises the provocative question: Are motile cilia Wnt-transducing organelles? If so, what response do they trigger and to what end?

To investigate the possibility of Wnt signaling in motile cilia, we used the embryonic skin of *X. tropicalis* (frog) embryos, a well-established model to study the development and function of mucociliary epithelia. Mammalian airway epithelia and the surface of *Xenopus* embryos are highly similar in cell composition and function, which has made *Xenopus* a model of choice for investigating novel regulatory mechanisms for motile cilia function and ciliopathy related phenotypes^17^.

Here, we reveal that motile cilia are Wnt signaling organelles that transduce a distinct Wnt ┫Pp1 response, with implications for the cell biology of Wnt signaling, cilia formation and function, and for new therapeutic avenues to ciliopathies.

## Results

### *X. tropicalis* motile cilia formation requires Wnt/Gsk3 signaling

**Fig. 1:**
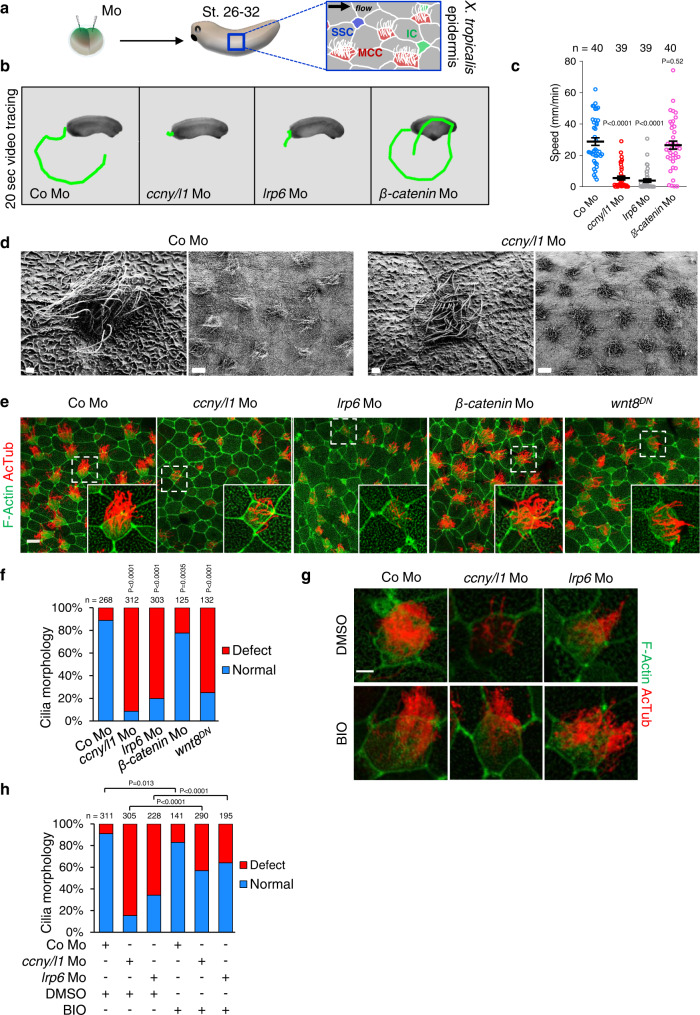
*X. tropicalis* motile cilia formation requires Wnt/Gsk3 signaling. **a** Microinjection and IF analysis scheme. MCC, multiciliated cells; IC, ionocytes; SSC, small secretory cells. **b** Visualization of motile cilia-driven embryo gliding video-tracked for 20 s in St. 28 *X. tropicalis* morphants (Mo). The distance moved by one representative embryo each is shown by green line traces. **c** Quantification of **b**. Data are biological replicates of four independent injection series displayed as means ± SEM. Unpaired two-tailed t-test used for statistical analyses. *n* = number of embryos analyzed per condition. **d** Representative SEM images of St. 28 *X. tropicalis* epidermis and MCC cilia at low and high magnifications. Scale bar 1 µm (high magnification) and 10 µm (low magnification). Note that *ccny/ccnyl1* morphant (Mo) MCCs display cilia defects. At least 3 embryos were analyzed for each set. **e** IF of *X. tropicalis* epidermis from embryos injected as indicated and stained with acetylated α-tubulin (AcTub) (cilia) and phalloidin for F-actin at St. 28. Scale bar 20 µm. **f** Quantification of **e**. Morphology of cilia in MCCs classified as normal or defect (shorter and fewer cilia). *n* = number of MCCs analyzed from >5 independent embryos from 3 experiments. Two-sided chi square test used for statistical analysis. **g** AcTub and phalloidin staining of St. 27 *X. tropicalis* morphants upon 6-bromoindirubin-3’-oxime (BIO, GSK3 inhibitor) treatment (60 µM) from St. 8 to St. 27. Scale bar 5 µm. **h** Quantification of **g**. MCC cilia rescue with BIO classified as normal or defect by comparison to control MCCs. Two-sided chi square test used for statistical analysis. *n* = number of MCCs analyzed from >6 independent embryos from 3 experiments. Where indicated, white dashed boxes are magnified in insets. Source Data files are available for figures **c**, **f** and **h**.

The *X. tropicalis* mucociliary epidermis develops after gastrulation, with postmitotic multiciliated cells (MCC) and multiciliary bundles beating in a polarized direction to generate fluid flow from head to tail of the embryo that removes pathogens and aids oxygenation (Supplementary Movie 1–4). Owing to directional cilia beating, *X. tropicalis* embryos slowly glide forward when placed on an Agarose gel layer (Supplementary Movie 5–9). Although ciliary gliding is variable between individuals and embryo batches, the mean gliding speed of a given batch provides a convenient and robust read-out for mucociliary function. We tracked the ciliary gliding movement of anesthetized tailbud stage embryos that were microinjected at two-cell stage with antisense morpholinos (Mo) targeting both *ccny* and *ccnyl1* (*ccny/l1*) (Fig. 1a, schematic). The specificity of these *ccny/l1* Mos was characterized previously^14,18^. Of note, adequately controlled Mos are a widely accepted and broadly used research tool in model organisms such as *X. tropicalis* harboring large stores of maternal RNAs that escape CRISP/Cas9-mediated genome editing^19^. All morpholino-induced phenotypes used in this study were validated by antibody staining and rescue experiments.

Strikingly, combined *ccny/l1* knockdown strongly reduced ciliary gliding (−79%) and ciliary beating (Fig. 1b, c; Supplementary Movie 2, 6). In situ hybridization confirmed that *ccny and ccnyl1* are both expressed in the *X. tropicalis* epidermis (Supplementary Fig. 1a). To confirm that this movement defect was related to Wnt signaling, we knocked down the Wnt co-receptor *lrp6* with a well-characterized morpholino^14,20^. Consistently, *lrp6* morphants also exhibited strongly reduced ciliary gliding (−85%) and beating (Fig. 1b, c; Supplementary Movie 3, 7). Interestingly, knockdown of *β-catenin* using a highly potent morpholino^21^, showed minor differences in cilia driven gliding and ciliary beating, despite embryos showing expected axial defects, including smaller heads (Fig. 1b, c; Supplementary Movie 4). To rule out that gliding defects in *ccny/l1* morphants were due to their slight developmental delay, we confirmed that earlier stage control embryos already display robust gliding movement (Supplementary Fig. 1b, c) and hence that developmental delay cannot account for reduced ciliary gliding in *ccny/l1* morphants. In addition, scanning electron microscopy (SEM) analysis of *ccny/l1* morphants revealed decreased number and length of cilia within individual MCCs (Fig. 1d). Motile cilia in morphants were also less polarized and less erect compared to controls. Immunofluorescence (IF) using the ciliary axoneme marker acetylated α-tubulin (AcTub) showed reduced ciliary density, length, and organization in *ccny/l1* morphants (Fig. 1e, f). While intercalation of MCCs into the epidermis was unaffected, morphants showed decreased MCC apical surface and F-actin levels, suggestive of reduced apical surface expansion, an F-actin-driven process^22^. Injection of mammalian *ccny/l1* mRNAs rescued the *ccny/l1* Mo-induced malformation of cilia in MCCs, confirming Mo specificity (Supplementary Fig. 1d, e). Similar cilia defects were observed in *lrp6* morphants and in embryos injected with mRNA encoding dominant-negative *wnt8* (*wnt8*^*DN*^)^23^, confirming ligand dependency (Fig. 1e, f). Knockdown of *β-catenin* with Mo had a smaller, yet significant effect on motile cilia formation with fewer and shorter cilia as reported^9^ (Fig. 1e, f), while it induced strong axial defects, confirming its efficacy (Supplementary Fig. 1f). We also corroborated the specificity of *ccny*, *ccnyl1*, and *lrp6* with a set of independent Mos targeting splice sites (*ccny/l1*^*SPL*^) and untranslated region (*lrp6*^*UTR*^), respectively. These new Mos reduced Lrp6 protein levels (Supplementary Fig. 1g) and *ccny*/*ccnyl1* RNA levels (Supplementary Fig. 1h, i), respectively, and impaired ciliary gliding (Supplementary Fig. 1j, k) and ciliogenesis (Supplementary Fig. 1l, m).

We ruled out that ciliogenesis defects in *ccny/l1* morphants are indirect consequences of altered cell fate specification or basal body localization of MCCs^24^. First, counting the number of MCCs showed that there was no significant change in *ccny/l1* morphants, while the number of MCCs in *β-catenin* morphants slightly increased (Supplementary Fig. 2a, b)^9^. Second, expression of *foxj1* and *foxa1*, which promote motile cilia formation and depend on canonical Wnt/*β-catenin* signaling^9–11,25^, was unaffected in *ccny/l1* morphants (Supplementary Fig. 2c, d). Third, since depletion of ionocytes in the larval epidermis can also lead to motile cilia defects^26^, we quantified these cells using the ionocyte marker ATP6V1A but found no change in *ccny/l1* morphants (Supplementary Fig. 2e). Fourth, since SEM images indicated reduced microvilli on goblet cells in *ccny/l1* morphants (Fig. 1d), we analyzed goblet cell composition and appearance but found them unchanged (Supplementary Fig. 2f). Fifth, co-injection into *ccny/l1* morphants of the basal body (BB) marker Centrin2-GFP with the BB polarity marker Clamp-RFP in combination with F-actin/phalloidin staining revealed a similar pattern of BB distribution and polarity as in controls, indicating that cilia defects observed in *ccny/l1* morphants are independent of assembly, polarity, or localization of BBs to the apical membrane (Supplementary Fig. 2g). Unlike *ccny/l1* morphants, a fraction of *lrp6* morphants showed fewer or irregular BBs (Supplementary Fig. 2h, i), consistent with a role of Lrp6 in planar cell polarity and BB assembly^27^.

Since CCNY/L1 mediate Wnt signaling by inhibiting GSK3-mediated phosphorylation of downstream targets^13^, we confirmed that the effects of *ccny/l1* and *lrp6* Mos were GSK3-dependent by rescuing motile ciliary defects in morphants with the small molecule GSK3 inhibitor 6-bromoindirubin-30-oxime (BIO)^28^ (Fig. 1g, h). Taken together, the results demonstrate that Wnt/Lrp6/Gsk3 signaling is required for *X. tropicalis* motile cilia formation. Since the magnitude of cilia malformation caused by Mos targeting *β-catenin* versus *lrp6*/*ccny/l1* did not compare, and since ciliary cell fate determination was unaffected, we conclude that the observed requirement for Wnt/Lrp6/Gsk3 signaling in ciliogenesis is mostly *β-catenin*-independent, which is corroborated by the data below.

### A Wnt ┫Pp1 signaling axis promotes motile ciliogenesis

**Fig. 2:**
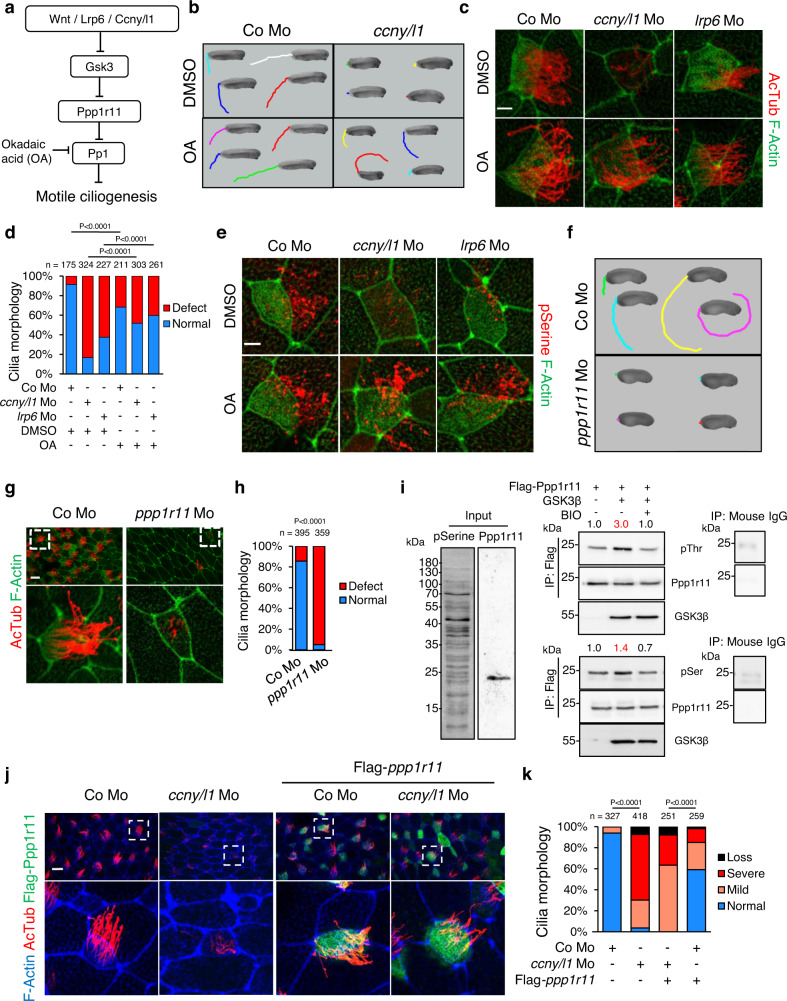
A Wnt ┫Pp1 signaling axis promotes ciliogenesis in motile cilia. **a** Wnt/Gsk3 signaling suppresses Protein phosphatase 1 (Pp1), by inhibiting Gsk3-mediated phosphorylation of Pp1 regulatory inhibitor subunit 11 (Ppp1r11). Okadaic acid (OA) inhibits Pp1 activity. **b** OA rescue of MCC-driven movements in *ccny/l1* morphants (Mo). St. 32 embryos were video-tracked for 20 s after treatment with 10 nM OA or DMSO. The distance moved by 4 representative embryos each is shown. **c** OA rescue of motile cilia in St. 26 *ccny/l1* morphants. *X. tropicalis* embryos were treated between St. 8-26 with OA. Scale bar 5 µm. **d** Quantification of **c**. Morphology of motile cilia was classified as normal or defect. **e** IF with pan phospho-Serine antibody (pSerine) in MCC cells of St. 27 morphants (Mo) *X. tropicalis* embryos treated with OA between St. 8-26. Scale bar 5 µm. **f** MCC-driven embryo movements of St. 27 control- and *ppp1r11* morphants video-tracked for 20 s. **g** IF for AcTub and phalloidin to visualize motile cilia morphology in St. 27 *ppp1r11* morphants. Scale bar 5 µm. **h** Quantification of **g**. Morphology of cilia was classified as normal or defect. **i** Immunoblot analysis of Ppp1r11 phosphorylation. Flag IP in 293T cell lysates after Flag-Ppp1r11 and GSK3β transfection ±BIO treatment, followed by immunoblot for pThr, pSer, Flag and GSK3. Mouse IgG IP as negative control. Numbers show normalization to control for pThr and pSer. **j** IF of MCCs for indicated proteins in St. 27 *ccny/l1* morphants co-injected with Flag-*ppp1r11* DNA. Scale bar 5 µm. **k** Quantification of **j**. Cilia morphology was classified as normal, mild (>half the length compared to control), severe (<half the length compared to control) or loss (no cilia). For statistical analysis ‘normal and mild’ and ‘severe and loss’ were pooled, respectively. Data information: Two-sided chi square test used for all statistical analyses. *n* = number of analyzed MCCs from >10 independent embryos from 3 experiments. White dashed boxes are magnified in lower panels. Source Data files are available for figures **d**, **h**, **i** and **k**.

We previously showed that Wnt/Ccnyl1/Lrp6 signaling inhibits PP1 via a GSK3β ┫ PPP1R2 ┫ PP1 axis during mouse sperm maturation^5^. Given the similarity between flagella and motile cilia, we asked if this signaling axis also operates in *X. tropicalis* motile cilia (Fig. 2a). To establish epistasis of PP1 with *ccny/l1* and *lrp6* in ciliogenesis, we conducted rescue experiments in morphant embryos by inhibiting PP1 by treatment with the phosphatase inhibitor okadaic acid (OA). OA markedly rescued impaired ciliary gliding in both *ccny/l1* and *lrp6* morphants (Fig. 2b; Supplementary Fig. 3a–e; Supplementary Movie 5–9). Moreover, OA treatment rescued the morphological cilia defects in *ccny/l1* and *lrp6* morphants (Fig. 2c, d).

Our model predicts that inhibition of Wnt/Gsk3 signaling derepresses Pp1 activity and thereby reduces protein phosphorylation. We therefore stained embryos with a pan-phospho-serine antibody (Ab), which revealed staining along the ciliary axoneme and towards the tips of motile cilia (Supplementary Fig. 3f). Phospho-serine was strongly reduced in *ccny/l1* and *lrp6* morphants, and this effect was efficiently rescued by OA treatment (Fig. 2e). As control, alkaline phosphatase abolished phospho-serine immunoreactivity, confirming Ab specificity (Supplementary Fig. 3g).

We sought to confirm the involvement of *ppp1r2* but the *X. tropicalis* orthologue is expressed only during later organogenesis^29^. However, its homolog *ppp1r11* may also be a relevant GSK3-regulated PP1 inhibitor because it acts redundantly with *ppp1r2* in sperm motility^30,31^. By whole-mount in situ hybridization, *ppp1r11* was broadly expressed in tailbud embryos, including in the mucociliary epithelium (Supplementary Fig. 3h). By IF, overexpressed Flag-tagged *ppp1r11* was highly enriched in the apical surface of MCCs and ciliary axoneme of motile cilia, suggestive of a ciliary function (Supplementary Fig. 3i). Indeed, injection of *ppp1r11* Mo targeting an mRNA splice site drastically reduced ciliary gliding of *X. tropicalis* tailbud embryos, while morphologically inducing only a mild axial defect or developmental delay (Fig. 2f; Supplementary Fig. 3j). Importantly, in *ppp1r11* morphants, cilia were almost absent and the few cilia remaining were defective (Fig. 2g, h). The cilia formation defect was once again accompanied by reduced apical surface expansion and apical actin levels. *Ppp1r11* morphants expectedly showed greatly reduced *ppp1r11* mRNA levels and injection of human *ppp1r11* mRNA effectively rescued cilia morphology, both results confirming morpholino specificity (Supplementary Fig. 3k–m). By immunoblot analysis, co-transfection of GSK3 increased phospho-serine and phospho-threonine levels in immuno-precipitated Ppp1r11 while BIO treatment reversed this effect, corroborating that Ppp1r11 is subject to GSK3 phosphorylation (Fig. 2i). Co-injection of *ppp1r11* DNA in *ccny/l1* morphants rescued their cilia defect, confirming that Ppp1r11 acts downstream of Ccny/l1 as in our model (Fig. 2j, k). In addition, treatment of *ppp1r11* morphants with the PP1 inhibitor OA partially restored cilia morphology, verifying that *ppp1r11* regulates MCC cilia via PP1 (Supplementary Fig. 3n, o). Collectively, the results support that an evolutionary conserved ciliary Wnt ┫Pp1 signaling axis promotes ciliogenesis in motile cilia (Fig. 2a; schematic).

### Motile cilia are Wnt signaling organelles

**Fig. 3:**
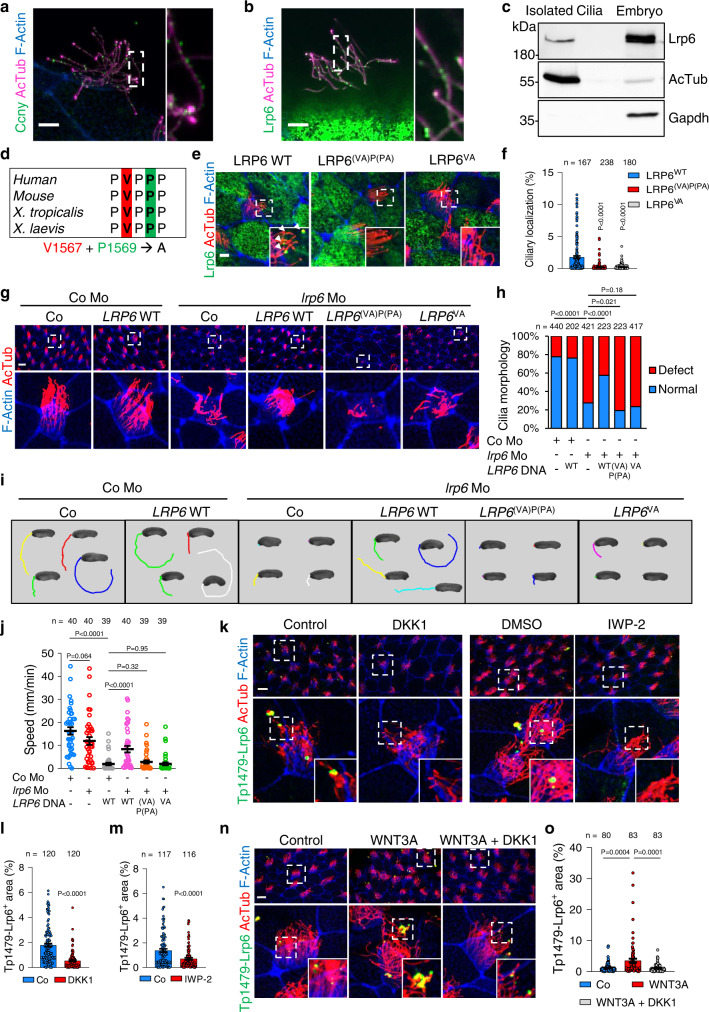
Ciliary Lrp6 is Wnt responsive. **a** High-resolution IF for endogenous Ccny, AcTub and F-Actin in St. 30 *X. tropicalis* MCCs. Scale bar 5 µm. **b** High-resolution IF for endogenous Lrp6, AcTub and F-Actin in St. 30 *X. tropicalis* MCCs. Scale bar 5 µm. **c** Immunoblot of purified motile cilia with indicated antibodies. **d** The conserved LRP6 CTS-like motifs V1567, or V1567 and P1569, were mutated in LRP6^VA^ and LRP6^(VA)P(PA)^ mutants, respectively. **e** Co-IF for Lrp6, AcTub and F-actin in St. 30 *X. tropicalis* MCCs upon LRP6 wild-type (WT), LRP6^(VA)P(PA)^ and LRP6^VA^ expression. Scale bar 5 µm. **f** Quantification of **e**. Data show expression areas of LRP6 relative to AcTub for individual MCCs, as means ± SEM. **g** IF for AcTub in St. 28 morphants co-injected with human *LRP6* DNA. Scale bar 5 µm. **h** Quantification of **g**. Cilia morphology classified as normal or defect compared to control. Two-sided chi square test for statistical analysis. **i** LRP6 WT or LRP6^VA^ and LRP6^(VA)P(PA)^ mutant rescue of gliding in *lrp6* morphants (Mo), video-tracked for 20 s at St. 28. **j** Quantification of **i**. Data are biological replicates of 4 independent injection series displayed as means ± SEM. *n* = number of embryos. **k** IF for ciliary phospho-Lrp6 (Tp1479) in St. 30 *X. tropicalis* MCCs 2 h after intra-chorionic membrane injections of DKK1 and IWP-2. Scale bar 20 µm. Quantification of DKK1 (**l**) and IWP-2 (**m**) injected samples from **k**. Data shows the expression area of Tp1479-Lrp6 relative to the AcTub area for individual MCCs, displayed as means ± SEM. ***n*** IF for endogenous Tp1479-Lrp6 in St. 30 *X. tropicalis* MCCs 2 h after intra-chorionic membrane injections of WNT3A and DKK1. Scale bar 20 µm. **o** Quantification of **n**. Data shows the expression area of Tp1479-Lrp6 relative to the AcTub area for individual MCCs, displayed as means ± SEM. Data information: Unpaired two-tailed t-test for statistical analyses unless indicated otherwise. *n* = number of MCCs analyzed from >10 embryos from 3 independent experiments. Source Data files are available for figures **c**, **f**, **h**, **j**, **l**, **m** and **o**.

We next addressed if *X. tropicalis* motile cilia harbor Wnt signaling components. High-resolution IF for Ccny and Lrp6 revealed the presence of both proteins in MCC motile cilia, appearing as distinct punctae along the ciliary axoneme (Fig. 3a, b). The Ccny and Lrp6 staining patterns were absent in their respective morphants, confirming specificity of the antibodies (Supplementary Fig. 4a, b). To corroborate Lrp6 localization to motile cilia, we purified motile cilia using the calcium chloride method^32^, and, by immunoblot analysis, detected Lrp6 protein (Fig. 3c). Gsk3 also localized to *X. tropicalis* motile cilia (Supplementary Fig. 4c).

Large transmembrane proteins (>40 kDa) require a ciliary targeting sequence (CTS) for translocation to the cilium^33^. Two common motifs are the VxP (VP) and the AxxxQ (AQ) motif^34,35^. Both motifs are present in human LRP6 but in *X. tropicalis* Lrp6 only the C-terminal **V**P**P** motif (aa 1567–1569) is conserved (Fig. 3d). We mutated this putative CTS as LRP6^VA^ and LRP6^(VA)P(PA)^ single- and double Alanine mutants, respectively (Fig. 3d). By IF, ciliary LRP6 localization was >10-fold reduced upon expression of either LRP6^VA^ or LRP6^(VA)P(PA)^ compared to WT (Fig. 3e, f). To link this finding to the above ciliogenesis defects, we performed rescue experiments. Only LRP6 WT but not LRP6^(VA)P(PA)^ or LRP6^VA^ reversed the ciliogenesis defects in *lrp6* morphants (Fig. 3g, h). Moreover, unlike WT LRP6, neither mutant reversed the *lrp6* Mo-induced ciliary gliding defect (Fig. 3i, j). Importantly, LRP6 WT and mutants showed equivalent protein expression (Supplementary Fig. 4d) and their Wnt signaling ability was comparable, as judged by *X. tropicalis* axis formation- and Wnt reporter (Topflash) assays (Supplementary Fig. 4e) and rescue of *lrp6* Mo-induced axial defects (Supplementary Fig. 4f, g). The results support that ciliogenic Lrp6 signaling proceeds within the cilium and that ciliary- and non-ciliary Lrp6 signaling can be uncoupled by mutating the CTS.

To confirm that ciliary Lrp6 responds to Wnt, we analyzed its activation using phospho-antibodies against Sp1490 and Tp1479, residues which are phosphorylated in response to Wnt ligand stimulation by GSK3 and CK1γ (Casein kinase 1 gamma) respectively, and thus indicate Wnt pathway activation^36^. In control embryos, Tp1479-Lrp6 and Sp1490-Lrp6 punctate signals were detected throughout the ciliary shaft of MCC cilia in around 50% and 25% of cells analyzed, respectively (Fig. 3k; Supplementary Fig. 5a, b). Ciliary Tp1479 and Sp1490 were reduced by a 2 h external treatment of embryos with two Wnt inhibitors: Recombinant DKK1 protein (Wnt antagonist) and IWP-2 (Porcupine small molecule inhibitor that blocks Wnt ligand secretion) (Fig. 3k–m; Supplementary Fig. 5b–d). This result indicates that unknown Wnt ligands in the epidermis trigger Lrp6 signaling in these motile cilia. Remarkably, treatment with WNT3A strongly induced ciliary Tp1479 and Sp1490 in MCCs, while DKK1 reversed this effect (Fig. 3n–o; Supplementary Fig. 5e, f). The specificity of ciliary Tp1479- and Sp1490 Lrp6 signals was confirmed in Lrp6 morphants (Supplementary Fig. 5g–j). Prominent localization to the ciliary tip (Fig. 3n; Supplementary Fig. 5e) prompted us to monitor co-localization of Wnt components with the exovesicle marker Tsg101^37,38^. However, Wnt components co-localized with the ciliary membrane rather than in TSG101-positive punctae (Supplementary Fig. 5k–n). Taken together, the results support that Lrp6 localizes to motile cilia of the epidermis and transmits a distinct ciliary Wnt ┫Pp1 cascade.

### Immediate response of motile cilia to Wnt stimulation

**Fig. 4:**
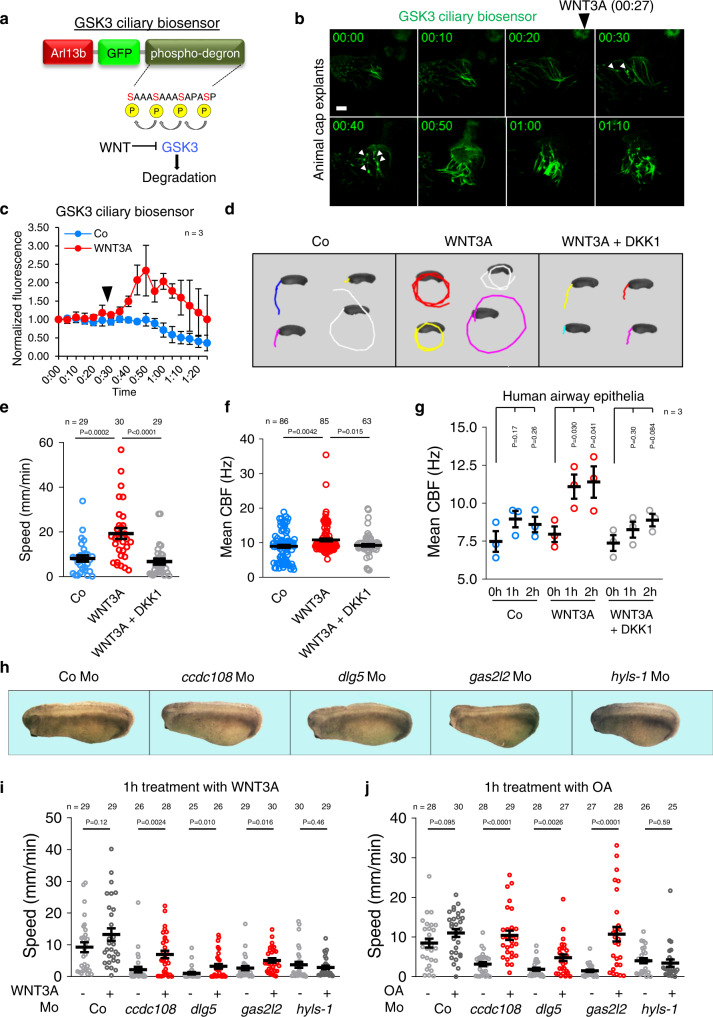
Wnt signaling stimulates ciliary beat frequency. **a** GSK3 sequentially phosphorylates serines (red), creating a phospho-degron that targets the biosensor for proteasomal degradation. Inhibition of GSK3 prevents proteasomal degradation and increases GFP levels. Fusion of sensor with mouse *Arl13b* targets the sensor to cilia. **b** Still images of GSK3 ciliary biosensor fluorescence acquired during live cell imaging of motile cilia in *X. tropicalis* animal cap explants at St. 30 equivalent. WNT3A recombinant protein was added after 27 min of imaging. GFP puncta (white arrowheads) are observed 3 min after WNT3A treatment. **c** Quantification of **b**. Data show the relative mean fluorescence of one MCC each after WNT3A and control treatment from 3 independent experiments. Arrowhead indicates begin of treatment. Fluorescence values were normalized to *t* = 0 in a given experiment and the mean was calculated from 3 experiments for each time-point and condition. **d** Ciliary gliding after 2 h of co-treatment with WNT3A ± DKK1, video-tracked for 20 s at St. 28. **e** Quantification of **d**. Data are biological replicates of 3 independent injection series displayed as means ± SEM. *n* = 30 embryos analyzed per condition. **f** High-speed time lapse imaging and CBF analysis of *X. tropicalis* MCCs after 2 h treatment with WNT3A ± DKK1. Data are biological replicates of 12 embryos from four independent batches each displayed as means ± SEM. *n* = total number of MCCs analyzed. **g** CBF analysis of human airway epithelial MCCs after 1 h and 2 h treatment with WNT3A ± DKK1. Data are biological replicates of three ALI inserts from one donor each displayed as means ± SEM. *n* = number of ALI inserts analyzed. **h** Phenotypes of indicated St. 28 ciliopathy morphants. Quantification of mucociliary gliding of ciliopathy morphants after 1 h treatment with WNT3A (**i**) or OA (**j**). Embryos were video-tracked for 20 s at St. 28. Data are biological replicates of 3 independent injection series displayed as means ± SEM. *n* = embryos analyzed per condition. Data information: Unpaired two-tailed t-test for all statistical analyses. Source Data files are available for figures **c**, **e**–**g**, **i** and **j**.

Sperm flagella deficient for Wnt signaling exhibit globally increased K48-linked protein ubiquitination due to GSK3 derepression^5^. Hence, we analyzed K48 ubiquitination in motile cilia by IF and observed punctate signals along the axoneme (Supplementary Fig. 6a). K48 ubiquitination was abolished by treatment with the GSK3 inhibitor BIO, suggesting that GSK3 regulates protein ubiquitination/degradation in motile cilia as it does in sperm flagella (Supplementary Fig. 6b). We took advantage of this insight to design a biosensor that permits live cell imaging of ciliary GSK3 activity. We used an established cytoplasmic GSK3 biosensor, in which four consecutive GSK3 phospho-degrons recognized by E3 polyubiquitin ligases are downstream of GFP. Phosphorylation of this biosensor by GSK3 targets GFP for rapid proteasomal degradation, while Wnt signaling inhibits GSK3 activity and stabilizes GFP^39^ (Fig. 4a, schematic). To target the biosensor to cilia, we fused it to Arl13b, injected it in *X. tropicalis* embryos, explanted ectodermal animal caps, and monitored the GFP signal in motile cilia by live cell imaging. Motile cilia bundles of untreated explants showed weak axonemal GFP fluorescence, with occasional individual brighter cilia, indicating successful ciliary targeting of the GSK3 biosensor (Fig. 4b). Strikingly, within 3 min of WNT3A addition, distinct bright axonemal punctae appeared that became more numerous by 10 min, to yield a bright homogeneous axonemal bundle fluorescent signal by 30 min (Fig. 4b, c; Supplementary Movie 10). The subsequent drop of the biosensor signal is likely an artifact caused by phototoxicity or photobleaching, since in control-treated embryos, baseline biosensor fluorescence also dropped (Fig. 4c; Supplementary Fig. 6c). A similar experiment in which whole embryos instead of animal caps were fixed after 30 min of treatment, confirmed that the ciliary GSK3 biosensor responds to WNT3A (Supplementary Fig. 6d, e). Taken together, these data support that motile cilia are (1) directly Wnt responsive, (2) exhibit GSK3-mediated protein degradation, consistent with the proposition of a ‘ciliary proteasome’^40^.

### Wnt signaling stimulates ciliary beating

The rapid Wnt response raised the possibility that ciliary Wnt signaling may not only promote ciliogenesis but also affect ciliary beating. To test this macroscopically, we treated *X. tropicalis* tadpoles with WNT3A and monitored ciliary gliding. Remarkably, a 2 h WNT3A pulse doubled the ciliary gliding speed of embryos while addition of DKK1 reversed the acceleration (Fig. 4d, e; Supplementary Movie 11–13). To monitor directly if WNT3A increases ciliary beat frequency (CBF), we employed high-speed time lapse imaging and SAVA analysis of MCCs to determine the CBF. WNT3A increased the mean CBF by ~20% and the effect was reversed by co-treatment with DKK1 (Fig. 4f, Supplementary Movie 14–16). While this increase in mean CBF is significant, it pales compared to the doubling of ciliary gliding speed (Fig. 4e), suggesting that WNT3A may also act by increasing the number, strength, or coherence of beating cilia.

To explore if Wnt stimulation of CBF is conserved in humans, we analyzed organotypic air-liquid interface (ALI) cultured primary human airway epithelial cells that form mucociliary epithelia^41^. A 1 h and 2 h WNT3A pulse induced the CBF by remarkable 40% and 43%, respectively (Fig. 4g; Supplementary Movie 17 and 18), in the range of what the best-established CBF accelerators, ATP and IL-1α, achieve^42^. This WNT3A effect was specific because it was completely reversed by DKK1 (Fig. 4g).

The evolutionary conservation of a CBF-stimulatory role prompted us to explore if Wnt ┫Pp1 signaling could be beneficial in human ciliopathies. Therefore, we injected previously characterized morpholinos that recapitulate ciliopathy- and fertility-associated diseases in *Xenopus* MCCs, including *ccdc108* (male infertility), *dlg5* (multi-organ congenital defects), *gas2l2* (primary ciliary dyskinesia), and *hyls-1* (perinatal hydrolethalus)^43–46^. *Ccdc108-*, *gas2l2-* and *hyls-1* morpholinos induced not only mucociliary deficiency but also mild axial defects (reduced head, increased belly) (Fig. 4h). Morpholino doses were adjusted so that they inhibited ciliary gliding but did not prevent ciliogenesis (Fig. 4i, j; Supplementary Fig. 6f, g). A 1 h pulse with either WNT3A or OA, although too short to noticeably accelerate gliding in control embryos, substantially (WNT3A) or fully (OA) rescued the embryo-gliding defects in *ccdc108* and *gas2l2* morphants, and partially rescued *dlg5*. Interestingly, both treatments failed to rescue *hyls-1* morphants, although their gliding impediment was less severe than that of e.g. *gas2l2* morphants (Fig. 4i, j; Supplementary Fig. 6h, i), supporting specificity of the phenotypic rescues. We note that unlike *hyls-*1 and *dlg5*, both *ccdc108* and *gas2l2* directly regulate the CBF^43,45^, suggesting a common mode of action that can be modulated by Wnt but whose precise mechanism remains to be established. Taken together, these results support that i) the Wnt ┫Pp1 signaling axis increases CBF, ii) that this feature is conserved, and iii) that activating the Wnt ┫Pp1 signaling axis improves ciliary performance in certain pathological contexts (Supplementary Fig. 6j, schematic).

## Discussion

The two main findings of this study are first that motile cilia are Wnt signaling organelles that transduce a Wnt ┫Pp1 response distinct from the canonical β-catenin cascade triggered across the non-ciliary plasma membrane. Thus, Wnts can elicit different responses in different plasma membrane compartments. Second, ciliary Wnt signaling is required for motile ciliogenesis, increases ciliary beat frequency, and improves ciliopathy-related CBF defects.

Conventionally, motile cilia are thought to exert mechanical force while primary cilia exhibit chemosensation. Yet, there are precedents for motile cilia bearing signaling receptors (e.g. human airway epithelial cells^15,47^) and exhibiting response to morphogens like Hh^16^, but the physiological significance remains unknown. Consistent with the idea of ‘chemosensory motile cilia’^47^, we demonstrate a motile ciliary Wnt ┫Pp1 response that is different from the canonical β-catenin cascade triggered across the plasma membrane. Our finding echoes the case of distinct Hh signaling responses across the primary cilium membrane vs. non-ciliary plasma membrane^48^. The results suggest that motile cilia may harbor a much richer chemosensory competence than is commonly thought.

We conclude that motile ciliary Wnt signaling is part of a multipronged Wnt-driven ciliogenesis program since Wnt also promotes motile cilia formation via canonical β-catenin signaling^8,10,11,49,50^. We uncoupled ciliary from canonical Wnt signaling i) by focusing on Ccny/l1, which as we show transduce mostly β-catenin-independent Wnt signaling, ii) by invoking the β-catenin-independent Wnt ┫Pp1 cascade, and iii) by introducing CTS-mutant Lrp6, which allowed us to specifically block ciliary- but not canonical Wnt/β-catenin signaling.

A consistent finding was reduced apical surface expansion and F-actin levels of MCCs in *ccny/l1*, *lrp6*, and *ppp1r11* morphants, which may relate to ciliogenesis defects. Since rescue of ciliogenesis- and gliding defects in *lrp6* morphants was achieved only by wt LRP6 but not by the LRP6 CTS mutants, the results support that apical surface expansion and F-actin levels are associated with ciliary Wnt signaling.

We find that in *X. tropicalis*, ciliary Wnt ┫Pp1 signaling is not only required for ciliogenesis but Wnt also stimulates ciliary beating, a process intimately linked to ciliogenesis and ciliopathies^1–3^. The Wnt effects on ciliary beating are of slow onset but more sustained than the fast onset-short duration effects (seconds-minutes range) induced by Ca^2+^ or serotonin^51,52^, likely due to different modes of action. Ciliary beating is regulated by the central microtubule pair, the radial spokes, and dynein motors^53,54^. Dynein activity is prominently regulated by phosphorylation, suggesting that kinases and phosphatases are key modulators of cilia motility^54^. PP1 is anchored to the flagellar axoneme^55^ and a trimeric complex of PP1, PPP1R7 and PPP1R11 blocks the catalytic activity of PP1^31,56^, which is sufficient to trigger sperm motility^57^. Our OA rescue experiments of ciliogenesis and motility support the central role played by PP1 and its regulation in cilia.

Supporting evolutionary conservation of motile ciliary Wnt signaling, we show accelerated ciliary beating of human airway epithelia upon Wnt stimulation. While the involvement of the PP1 axis remains to be confirmed in human airway epithelia, we note that inhibiting Wnt secretion in *Porcupine*-deficient mouse airway epithelia impairs motile ciliogenesis^58^. Likewise, acute inhibition of Wnt secretion reduces the ability of multiciliated mouse oviduct epithelium to transport embryos and, interestingly, in a β-catenin-independent manner^59^. Moreover, PP1 inhibition reverses alcohol-induced motile ciliary dysfunction^60^. Together with our previous finding in sperm^5^ we therefore propose that direct ciliary Wnt ┫Pp1 signaling acts as common direct regulator of cilia function in flagella and motile cilia. Future analyses should confirm the involvement of GSK3 and PP1 in this context. Overall, the mechanistic insight of our study and the discovery that Wnt signaling stimulates ciliary beating and improves CBF deficiency in ciliopathy models, may aid in the understanding of ciliopathies and inspire novel therapeutic interventions. *Note added in proof*: While this paper was in production, we reported that primary cilia are also WNT ┫PP1 signaling organelles^61^, indicating that the three main ciliary classes—flagella^5^, motile cilia (this work), and primary cilia—employ ciliary Wnt signaling for ciliogenesis.

## Methods

### *X. tropicalis* microinjection and treatments

*X. tropicalis* frogs were obtained from Nasco, the National Xenopus Resource (NXR) and the European Xenopus Resource Centre (EXRC). All animal experiments were approved by the state review board of Baden-Württemberg, Regierungspräsidium Karlsruhe, Germany (permit number 35-9185.81/G-141/18). Federal and institutional guidelines and regulations were followed. Developmental stages were determined according to Nieuwkoop and Faber (Xenbase). Statistical analysis to determine sample size, sex- and gender-based analyses, and randomization of injection were not applicable in this context. In vitro fertilization and culture of embryos were performed as previously described^62^. Antisense morpholino oligonucleotides (Mo) were obtained from GeneTools and microinjected using the Harvard Apparatus microinjection system. Morpholinos targeting *ccny* (10 ng per embryo^14^), *ccny*^*SPL*^ (10 ng per embryo; 5’-ATGTTTCCACAGTACGAGAAAAACG-3’), *ccnyl1* (10 ng per embryo^14^), *ccnyl1*^*SPL*^ (10 ng per embryo; 5’-TTCATTGCAGATTTTCACGATGAGC-3’), *lrp6* (5 ng per embryo^20^), *lrp*^*UTR*^ (5 ng per embryo; 5’-GCTCAATGCTCCCCCGTAAGCCAGC-3’), *β-catenin* (10 ng per embryo^21^), *ppp1r11* (30 ng per embryo; 5’-AGAGCGGTGTTCCTGTTACGGGTAA-3’), *ccdc108* (10 ng per embryo^43^), *dlg5* (10 ng per embryo^44^), *gas2l2* (5 ng per embryo^45^), *hyls-1* (10 ng per embryo^46^) and standard control (up to 30 ng per embryo) as well as *wnt8*^*DN*^ mRNA (500 pg per embryo) and membraneRFP (mbRFP) mRNA (300 pg per embryo) were microinjected animally 5 nl per each embryonic blastomere. In *ccny/l1* DKD, 10 ng of each Mo were co-injected either alone, or with human *CCNY-*Flag (250 pg per embryo)^63^ and mouse *Ccnyl1*-Flag mRNA (250 pg per embryo)^14^ for rescue experiments. BB marker pCS2-gfp-drCentrin 2 (100 pg per embryo) was co-injected with VF10-RFP-Clamp (150 pg per embryo) and indicated morpholinos. pCS2-gfp-drCentrin 2 was a kind gift from Wieland Huttner, and VF10-RFP-Clamp was generously provided by Gerd Walz. *X. tropicalis* Flag-*ppp1r11* mRNA was injected at a concentration of 200 pg per embryo to investigate ciliary localization, while human *ppp1r11* mRNA without Flag-Tag was injected at 25 pg and 50 pg for *ppp1r11* Mo rescue experiments. In Fig. 2j, *ccny/l1* morphants were rescued with 50 pg of Flag-*ppp1r11* DNA. Human *LRP6* WT, *LRP6*^(VA)P(PA)^ and *LRP6*^VA^ DNA were injected at a concentration of 50 pg per embryo. For GSK3 ciliary biosensor experiments, 100 pg biosensor DNA was co-injected with 100 pg of Arl13b-mKate2 DNA. Animal cap explants were dissected at St. 8 and cultured until St. 30 for live cell imaging. WNT3A recombinant protein (R&D Systems; Cat#5036-WN-010) was added at a concentration of 2 µg/ml. In Wnt stimulation of whole embryos, WNT3A was added for 30 min before fixation. Embryos were cultured until St. 28-32 unless indicated otherwise. BIO (Cayman Chemical, Cat#13123) rescue was performed from St. 8 to St. 28 by adding 60 µM BIO to the embryo culture medium. Two different modes were applied for treatment with OA (Cell signaling technology, Cat#5934): 10 nM OA in DMSO (Sigma; Cat#D2650-100ML) was added from St. 26 to St. 32 to analyze movements of epidermal MCCs and for ciliopathy Mo rescue experiments the embryos were analyzed after 1 h, at St. 28. Cilia morphology was analyzed after treatment with 10 nM OA from St. 8 to St. 26. Control groups were treated with DMSO.

### Ciliary gliding assay

Embryo tracking on fresh 1% agarose gels was performed using the Serial Images function of AxioCam MRc 5 microscope (Zeiss). 10 embryos per condition were transferred to 0.02% Tricaine/MS222 (Sigma) for anesthesia before they were placed on a 1% agarose gel where 20 images were taken, one image per sec. FIJI software was used to generate time-lapse movies of 5 frames/sec. Embryos were tracked with the ‘manual tracking’ function and the distance traveled was calculated as mm/min for each embryo in Microsoft Excel. For rescue experiments, the same embryos were tracked before and after treatment with DMSO/OA. Four embryos were chosen as representative images per figure for each condition and the background was adjusted using the Magnetic Lasso tool in Adobe Photoshop CS6. Quantifications were performed for the indicated number of embryos from 3 or more independent injections.

### Chorionic membrane injections

St. 25 embryos were injected in the chorionic membrane with 5–10 nl of recombinant human DKK1 (3 ng) (Peprotech; Cat#120-30-10UG), IWP-2 (12 ng) (Sigma; Cat#686770-61-6) or recombinant human WNT3A (2 ng; Cat#5036-WN-010). For WNT3A co-injection 2 ng DKK1 was used. As controls, 0.1% BSA in PBS or (0.1% BSA, 0.1 mM EDTA (Sigma), 0.5% (w/v) CHAPS (MP Biomedicals), 0.5% (w/v) DMSO) in PBS were used, respectively. After 2 h incubation, embryos were manually hatched and fixed in 4% PFA for immunostaining.

### Motile cilia isolation

The deciliation procedure was adapted from Werner and Mitchell, 2013^32^. Embryos were cultured until St. 30. Between 70 and 100 embryos were deciliated in 75 mM CaCl_2_, 0.02% NP-40 (Sigma) in 1/19 MR for 2 min or until no cilia driven movements were observed, and then the buffer containing motile cilia was centrifuged (15.000 g, 7 min). The cilia pellet was reconstituted in 20 µl of NOP + buffer (20 mM Tris-HCl pH 7.5, 150 mM NaCl, 2% Triton X-100, 0.2% DTT, and cOmplete Protease Inhibitor Cocktail (Roche; Cat#11697498001)). NuPAGE LDS buffer (Thermo Scientific; Cat#NP0007) (+50 mM DTT) was added in a 1:4 ratio and samples were heated at 70 °C for 10 min before immunoblotting.

### LRP6 mutagenesis, and cloning of Flag-Ppp1r11 and GSK3 ciliary biosensor

Mutagenesis of *LRP6*^VA^ and *LRP6*^*(VA)P(PA)*^ was performed by PCR-amplifying full length *LRP6* with Phusion^TM^ DNA polymerase using primers designed to mutate selected amino acids into alanines: *LRP6*^VA^ forward: 5’-GATTCAGAACCTGCGCCCCCACCTCCCACACCC-3’; *LRP6*^*(VA)P(PA)*^ forward: 5’-GATTCAGAACCTGCGCCCGCACCTCCCACACCC-3’; common reverse: 5’-GGGTGTGGGAGGTGCGGGCGCAGGTTCTGAATC-3’. GoTaq (Promega; Cat# M7841) DNA polymerase was used to amplify full-length Ppp1r11 using cDNA obtained from whole *X. tropicalis* embryos (Forward: 5’-TAAGCAGAATTCATGGCAGAATCCTCCGGG-3’; Reverse: 5’-TGCTTACTCGAGTTAGTGTTGCATGCTGCC-3’). Where indicated, N-terminal Flag tags were added via forward primers (Forward: 5’-TAAGCAGAATTCATGGATTACAAGGATGACGACGATAAGGCAGAATCCTCCGGGCCG-3’). The GSK3 ciliary biosensor (pArl13b-GSK3) was generated as follows: mouse Arl13b CDS without stop codon and the coding sequence of a linker peptide (Glycine-Glycine-Glycine-Glycine-Serine) were inserted upstream of the GFP coding sequence in a pCS2-GFP-GSK3-MAPK-Flag backbone plasmid (kind gift from De Robertis lab, see ref. ^39^). The mArl13b-linker part was amplified from E13.5 mouse whole brain cDNA by using the forward primer 5’-TTGCAGGATCCGCCACCATGTTCAGTCTGATGGCCAACTG-3’, and the reverse primer 5’-CTTGCTCACTGATCCTCCTCCTCCTGAGATCGTGTCCTGAGCAT-3’. The Linker-pCS2-GFP-GSK3-MAPK-Flag part was amplified from the pCS2-GFP-GSK3-MAPK-Flag backbone with the forward primer 5’-CGATCTCAGGAGGAGGAGGATCAGTGAGCAAGGGCGAGGAGCT-3’ and the reverse primer 5’-CAGTTGGCCATCAGACTGAACATGGTGGCGGATCCTGCAA-3’. Gibson assembly was performed according to the protocol from the NEB Gibson assembly kit (#E5510). Positive clones were picked and confirmed by Sanger-sequencing.

### Scanning electron microscopy

Scanning electron microscopy (SEM) was performed as previously described^64^. Briefly, whole St. 28 *X. tropicalis* embryos were fixed with EM fixation buffer (4% Formaldehyde, 2% Glutaraldehyde, 10 mM Cacodylate, 1 mM CaCl_2_, 1 mM MgCl_2_, pH 7.2) for 16–24 h at 4 °C. Embryos were then washed several times and post-fixed with 2% osmium tetroxide, 1.5% potassium ferrocyanide for 1 h. Following post-fixation, samples were washed and dehydrated with an ascending series of ethanol and pure acetone before critical point drying. The samples were then sputter-coated with an 80% gold, 20% palladium alloy and examined with an ULTRA 55 field-emission scanning electron microscope (ZEISS).

### Whole-mount in situ hybridization (WISH)

WISH was performed as previously described using digoxigenin (DIG)-labeled probes^62^. In brief, embryo were fixed in MEMFA, dehydrated in MeOH and incubated over night at 67 °C with 500ng-1µg of the DIG labeled probes described below. The anti-DIG antibody (Sheep polyclonal anti-digoxigenin-AP, Roche, Cat#11093274910) was diluted 1:2500 and incubated for 4 h at room temperature. Expression was visualized using BM purple AP substrate (Sigma-Aldrich, 11442074001).

A PCR-based approach was conducted to generate ORF templates for antisense/sense RNA probes against *ccny* and *ccnyl1*. The first PCR reaction generated 827 bp and 960 bp templates from *X. tropicalis ccny* and *ccnyl1* CDS mRNA, respectively (*ccny* forward: 5’-GAGCAGGACATAAGCAGAGAGG-3’, reverse: 5’-GCCTCAAGTTTACATGCACGATCC-3’; *ccnyl1* forward: 5’-AACACCGTGACCTGTTGCGTG-3’; reverse: 5’-GGCAGCCTTAGATAGGTCCTTG-3’). In a second PCR reaction the T7 promotor sequence (5’-CTAATACGACTCACTATAGGG-3’) was added to the primers (sense: forward primer; antisense: reverse primer) to generate templates for in vitro transcription. In vitro transcription was performed as previously described^65^. *Ppp1r11* WISH probes were generated after cloning the *X. tropicalis ppp1r11* PCR product into the pCS2+ plasmid. pCMV-Sport-*foxa1* and pCS2 + -*foxj1 X. laevis* probes were a kind gift from Martin Blum and linearized with Sal1 and Stu1, respectively, and used for WISH in *X. tropicalis*. Linearization and in vitro transcription were performed as previously described^65^. Representative images were obtained by AxioCam MRc 5 microscope (Zeiss) and the background was adjusted using the Magnetic Lasso tool in Adobe Photoshop CS6.

### Immunofluorescence

*X. tropicalis* embryos at St. 26–30 were fixed in 4% Paraformaldehyde for 3 h at RT except for Atp6v1a and Itln1 staining, which employed Dent’s Fix (4:1 Methanol:DMSO) for 3 h at −20 °C. After several washing steps, the samples were incubated in PPBSTB (0.1% Triton X-100, 2% BSA in PBS) for 1 h before the primary antibodies were added in a 1:300 dilution overnight at 4 °C (Rabbit monoclonal anti-LRP6, Cell Signaling Technology, Cat#2560; Mouse monoclonal anti-acetylated alpha tubulin, Sigma-Aldrich, Cat#T7451; Rabbit polyclonal anti-CCNY, homemade,^14^; Rabbit monoclonal anti-GSK3α/β, Cell Signaling Technology, Cat# 5676; Rabbit polyclonal anti-Phospho-LRP6 (S1490), Cell Signaling Technology, Cat#2568S; Rabbit polyclonal anti-Phospho-LRP6 (T1479), homemade,^36^; Mouse monoclonal anti-FLAG M2, Sigma Aldrich, Cat#F3165; Mouse monoclonal anti-Phosphoserine (Clone 4A4), Millipore, Cat#05-1000; Rabbit polyclonal anti-ATP6V1A, Abcam, Cat#ab137574; Rabbit monoclonal anti-Ubiquitin, Lys48-Specific, clone Apu2, Millipore, Cat#05-1307; Rabbit polyclonal anti-ITLN1, Proteintech, Cat#11770-1-AP; Rabbit monoclonal anti-Acetyl-α-Tubulin (Lys40), Cell Signaling Technology, Cat#5335T; Mouse monoclonal anti-TSG101 (4A10), Abcam, Cat#ab83). Secondary antibodies (Donkey polyclonal Anti-Mouse IgG, Alexa Fluor 647, Jackson ImmunoResearch, Cat#715-605-151; Donkey polyclonal Anti-Mouse IgG, Alexa Fluor 488, Jackson ImmunoResearch, Cat#715-545-150; Donkey polyclonal Anti-Rabbit IgG, Alexa Fluor 647, Jackson ImmunoResearch, Cat#711-605-152; Goat polyclonal Anti-Rabbit IgG, Alexa Fluor 488, Invitrogen, Cat#A11008) were diluted 1:500 in PBSTB and applied for 3 h at RT together with 1:1000 Phalloidin-iFluor 405 or −488 Reagent (Abcam; Cat#ab176752 and Cat#ab176753, respectively). Small embryo pockets were prepared by putting 2 layers of isoelectric tape on a glass slide and cutting rectangles with a scalpel. One embryo was mounted in each pocket.

### Immunoprecipitation

Pull-down of Ppp1r11 was performed in HEK293T cells 48 h after reverse transfection of Flag-Ppp1r11 ± GSK3β. BIO treatment (1 µM) was performed for 2 h at 37 °C prior to harvest. Cultured cells were resuspended in Triton lysis buffer (20 mM Tris HCl, 150 M NaCl, 1% Triton X-100, 1 mM EDTA, 1 mM EGTA, 1 mM b-glycerolphosphate, 2.5 mM sodium pyrophosphate and 1 mM sodium orthovanadate). After cell lysis, 30 min incubation on ice, centrifugation and concentration measurement, the samples were incubated with Protein A/G PLUS-Agarose (Santa Cruz; sc-2003) and Flag antibody (Mouse monoclonal anti-FLAG M2, Sigma Aldrich, Cat#F3165) 1 :250 for 3 h. As a negative control for IP, mouse γ-globulin was used 1 :250. Lysates were washed 3x in lysis buffer then heated at 70 °C in NuPAGE LDS buffer (Thermo Scientific; Cat#NP0007) supplemented with 50 mM DTT for subsequent immunoblotting.

### Immunoblotting

*X. tropicalis* embryos were harvested at indicated stages, homogenized in 20 µl per embryo of NOP + lysis buffer (20 mM Tris-HCl pH 7.5, 150 mM NaCl, 2% Triton X-100, 0.2% DTT, and cOmplete Protease Inhibitor Cocktail (Roche; Cat#11697498001)). Lysates were cleared with CFC-113 (Honeywell, 34874) and centrifugation (18,800 × *g*, 10 min at 4 °C). After boiling at 95 °C for 5 min with NuPAGE LDS Buffer (+50 mM DTT), 0.5–1 embryos per lane were loaded for SDS-PAGE analysis. *X. tropicalis* lysates and immunoprecipitation samples were separated on polyacrylamide gels, transferred to nitrocellulose and blocked with 5% skim-milk powder or 5% BSA in Tris-buffered saline with 0.1% Tween-20 (TBST) for 1 h at room temperature. Primary antibody was diluted 1:1000 in blocking 1% buffer and incubated overnight at 4 °C. Membranes were incubated with peroxidase-linked secondary antibodies diluted 1:5000 for 1 h at RT and then treated with Supersignal West Pico solution (Thermo Scientific; Cat#34580). Images were acquired on an LAS-3000 system (Fuji Film). Primary antibodies used for immunoblotting: Rabbit monoclonal anti-GAPDH, Cell Signaling Technology, Cat#2118S; Rabbit monoclonal anti-LRP6, Cell Signaling Technology, Cat#2560; Mouse monoclonal anti-acetylated alpha tubulin, Sigma-Aldrich, Cat#T7451; Mouse monoclonal anti-GSK3β, BD Biosciences, Cat#610201; Mouse monoclonal anti-FLAG M2, Sigma Aldrich, Cat#F3165; Mouse monoclonal anti-Phosphoserine (Clone 4A4), Millipore, Cat#05-1000; Mouse monoclonal anti-Phosphothreonine (Q7), Qiagen, Cat#37420. Secondary antibodies: Goat anti-mouse IgG (H + L) HRP, Jackson ImmunoResearch, Cat#115-035-146; Goat anti-rabbit IgG (H + L) HRP, Jackson ImmunoResearch, Cat#111-035-144 and Donkey anti-Goat IgG-HRP, Santa Cruz, Cat#sc-2020.

### Topflash

*X. tropicalis* embryos were co-injected with 80 pg TOPFlash and 20 pg Renilla per embryo at St. 2 and 5 embryos (St. 11) were lysed in 33 µl of Passive lysis buffer for each replicate. Luciferase activity was measured with the Dual luciferase reporter assay system (Promega; Cat#E1960) according to the manufacturer’s protocol. Firefly luminescence (TOPFlash) was normalized to Renilla.

### Real-time quantitative PCR

*X. tropicalis* embryos were harvested at St. 22 and lysed in 1 ml of TRizol (Ambion, 18914101). Extraction and precipitation of RNA were performed following the manufacturer’s instruction. Extracted mRNA was transcribed to cDNA using random hexamer primers. PCR was performed on a Roche Light Cycler 480 using the Universal ProbeLibrary system (*ppp1r11*: Forward: 5’-TGCGAAAGAGAAAGCCTGAT-3’, Reverse: 5’-TCTTGGCTTCTCATAGATACAGCA-3’, UPL probe #39; *ccny*: Forward: 5’- ATCTCAGACCGATGTACGAGAA-3’, Reverse: 5’-ACGAGCTGTATTTTCGCCGT-3’, UPL probe #37; *ccnyl1*: Forward: 5’- CGGGAGTTCCCTGTTGATTTT-3’, Reverse: 5’-TTGGTAAGCTGCCCAGGAGA-3’, UPL probe #44).

### Live cell imaging

*X. tropicalis* embryos were injected with GSK3 ciliary biosensor (100 pg per embryo) and cultured until St. 8 and animal cap explants were dissected. Eight explants were seeded with the apical side on Lab-Tek II 4-well chambers coated with 1 mg/ml Concanavalin A (Sigma). The explants were cultured in Steinberg’s Solution until St. 30. Imaging was performed on an LSM780 confocal microscope (Zeiss). Z-stacks were taken at 0.38 µm steps at 5 min time intervals for a total of 30 time points. After the 6^th^ time point, at 0:27, 2 µg/ml WNT3A recombinant protein or control buffer (0.1% BSA, 0.1 mM EDTA, 0.5% (w/v) CHAPS, 0.5% (w/v) DMSO in PBS) were added.

### Image acquisition and analysis

A Zeiss LSM 700 microscope was used for imaging of IF samples. The pinhole was set to 1 Airy unit at maximal optical resolution before gain and offset were calibrated. Fluorescent signal intensities were within the linear range of detection. For all IF image analyses, ImageJ software was used. For high resolution IF, a 40x silicone immersion objective (numerical aperture: 1.25) was used at the Nikon AX confocal microscope. Pixel size was set to 100 nm and z sections were obtained at the ventral rim of the embryo to reduce background signal. Images were deconvolved using Nis-Elements 3D Deconvolution. Quantification of the ciliogenesis defects in *X. tropicalis* MCC cells was performed by immunostaining against acetylated tubulin and phalloidin to identify the ciliary axoneme and MCC border, respectively. MCC motile cilia were classified as ‘Normal’ or ‘Defect’ (showing lower cilia density and shorter cilia) as well as ‘Mild’ (>half the length compared to control), ‘Severe’ (<half the length compared to control) and ‘Loss’ (no cilia). Quantifications were performed on the indicated number of embryos from three to six independent experiments. Cell fate changes in *X. tropicalis* embryos were examined by calculating the ratio of MCC or Ionocytes to total cells. pLrp6 stainings as well as LRP6 WT, LRP6^VA^ and LRP6^(VA)P(PA)^ stainings were quantified by measuring their IF-positive areas and then normalizing to the AcTub^+^ area for each MCC using Fiji (ImageJ). Each data point represents one ratio. For the GSK3 ciliary biosensor live cell imaging, the mean signal intensity was measured for one MCC per experiment and time-point. The mean signal intensity for each MCC was normalized to that of the first time point (*t* = 0) in the representative graph. In fixed ciliary GSK3 biosensor-injected embryos, one full image per embryo was measured using Fiji (ImageJ). Ciliary GSK3 mean intensity was normalized to Arl13b-mKate2 mean intensity in the latter.

Images for phenotypical analyses were taken with the AxioCam MRc 5 microscope (Zeiss). Embryos were scored blind and data are representative images from 2 or more independent experiments. Representative embryos were selected using Magnetic Lasso tool of Adobe Photoshop CS6. Background was adjusted uniformly for presentation. Phenotypical features were analyzed by comparison to control embryos and classified and ‘normal’ and ‘defect’ (showing DV/AP patterning defects).

### High-speed time lapse imaging & SAVA analysis

Embryos were cultured until St. 28 and treated with 2 µg/ml WNT3A ± 2 µg/ml DKK1 recombinant protein or control buffer for 2 h. For imaging, live embryos were placed into pockets on a cover slip made with 2 stripes of isoelectric tape and covered with a second coverslip to prevent movement immediately before imaging. High-speed time lapse sequences of *X. tropicalis* MCCs were recorded on a Nikon Ti2 inverted microscope equipped with a 20x water immersion objective (NA 0.8) and differential interference contrast to visualize cilia motion. Acquisition was performed by Nis-Elements 5.2 on a sCMOS camera Andor sNEO in binning 2 resulting in a pixel size of 0.43 µm. The following settings were chosen to achieve 993 fps: RAM capture was used and a small region of interest (350 × 100 pixel) in the center was read out using the rolling shutter for 2–4 s. At least 12 embryos from 4 independent batches and experiments were analyzed. 5 regions per embryo, displaying 2–4 MCCs were imaged. One second (993 frames) was converted to avi format from each raw video file using FIJI (ImageJ) before CBF analysis with the SAVA software^66^. One ROI was defined for each MCC and the mean CBF was measured for each ROI.

### Human respiratory epithelial cells

Human respiratory epithelial cells were obtained from the middle turbinate of healthy donors by nasal brush biopsies (Engelbrecht Medicine and Laboratory technology) and suspended in cell culture medium. After pre-culturing in rat collagen-coated cell culture flasks, the cells were processed to air-liquid interface (ALI) cultures as previously described^41,67^. PneumaCult™-Ex Medium (StemcellTM) was used for the proliferation phase and PneumaCult™-ALI Medium (StemcellTM) supplied with 1% Antibiotic-Antimycotic 100x (Gibco) for the ALI cultures. For CBF analysis, individual ALI inserts were washed with PBS and 200 µl of PneumaCult™ -ALI Medium (StemcellTM) were applied to the apical side for prospective treatments. After a 20 min adjustment phase at the microscope, CBF values were directly measured with the SAVA software (whole field analysis, 512 frames with 200 frames per sec) for 5 min in 10 s intervals before treatment (t_0_), after 1 h and after 2 h of apical treatment with 1 µg/ml WNT3A ± 1 µg/ml DKK1 recombinant protein or control buffer. Between measurements, the plate was placed back into the incubator. A linear fit was calculated for each time-point and condition and the first measurement (0:10) was chosen for further comparison and statistical analyses.

### Data analysis

Sample sizes are reported in each figure legend. All statistical analyses were conducted using Graphpad Prism. Means between two groups were compared using two-tailed unpaired Student’s *t* test, or with two-sided Chi square test. Results are displayed as arithmetic mean ± standard error of mean (SEM). Where indicated, results are shown as fold change vs. controls. Statisticsare indicated as *p* values.In all ciliary gliding assays, embryos that woke up from anesthesia were excluded, and significant outliers were identified and eliminated by Grubb’s test.

### Reporting summary

Further information on research design is available in the Nature Portfolio Reporting Summary linked to this article.

## Supplementary information

Supplementary Information

Description of Additional Supplementary Files

Supplementary Movie 1

Supplementary Movie 2

Supplementary Movie 3

Supplementary Movie 4

Supplementary Movie 5

Supplementary Movie 6

Supplementary Movie 7

Supplementary Movie 8

Supplementary Movie 9

Supplementary Movie 10

Supplementary Movie 11

Supplementary Movie 12

Supplementary Movie 13

Supplementary Movie 14

Supplementary Movie 15

Supplementary Movie 16

Supplementary Movie 17

Supplementary Movie 18

Reporting Summary

## Data Availability

NCBI Reference Sequences used for cloning were as follows: Human lrp6 BC143725.1, *Xenopus tropicalis* ppp1r11: NM_001004815.1, Human ppp1r11: NM_021959.3. Xenbase (https://www.xenbase.org/entry/) was used to gather information about morpholinos and expression patterns. No third party datasets were analyzed in this study. Further information on research design is available in the Reporting Summary. All original data and raw microscopy images are available from the corresponding author upon request. Source data are provided with this paper.

## Source data

Source Data
