# Supplementary material for: Mucociliary Wnt signaling promotes cilia biogenesis and beating
Source: Nat Commun. 2023 Mar 6;14:1259. doi: 10.1038/s41467-023-36743-2 (PMC9988884; doi:10.1038/s41467-023-36743-2)
Supplement: Supplementary file 1 — Supplementary Information [file 41467_2023_36743_MOESM1_ESM.pdf]

Supplementary information:

Mucociliary Wnt signaling promotes cilia biogenesis and beating

Carina Seidl[1], Fabio Da Silva[1], Kaiqing Zhang[1], Kai Wohlgemuth[2], Heymut Omran[2], and Christof Niehrs[1,3]*

Supplementary Figures 1-6

Supplementary Figure 1.

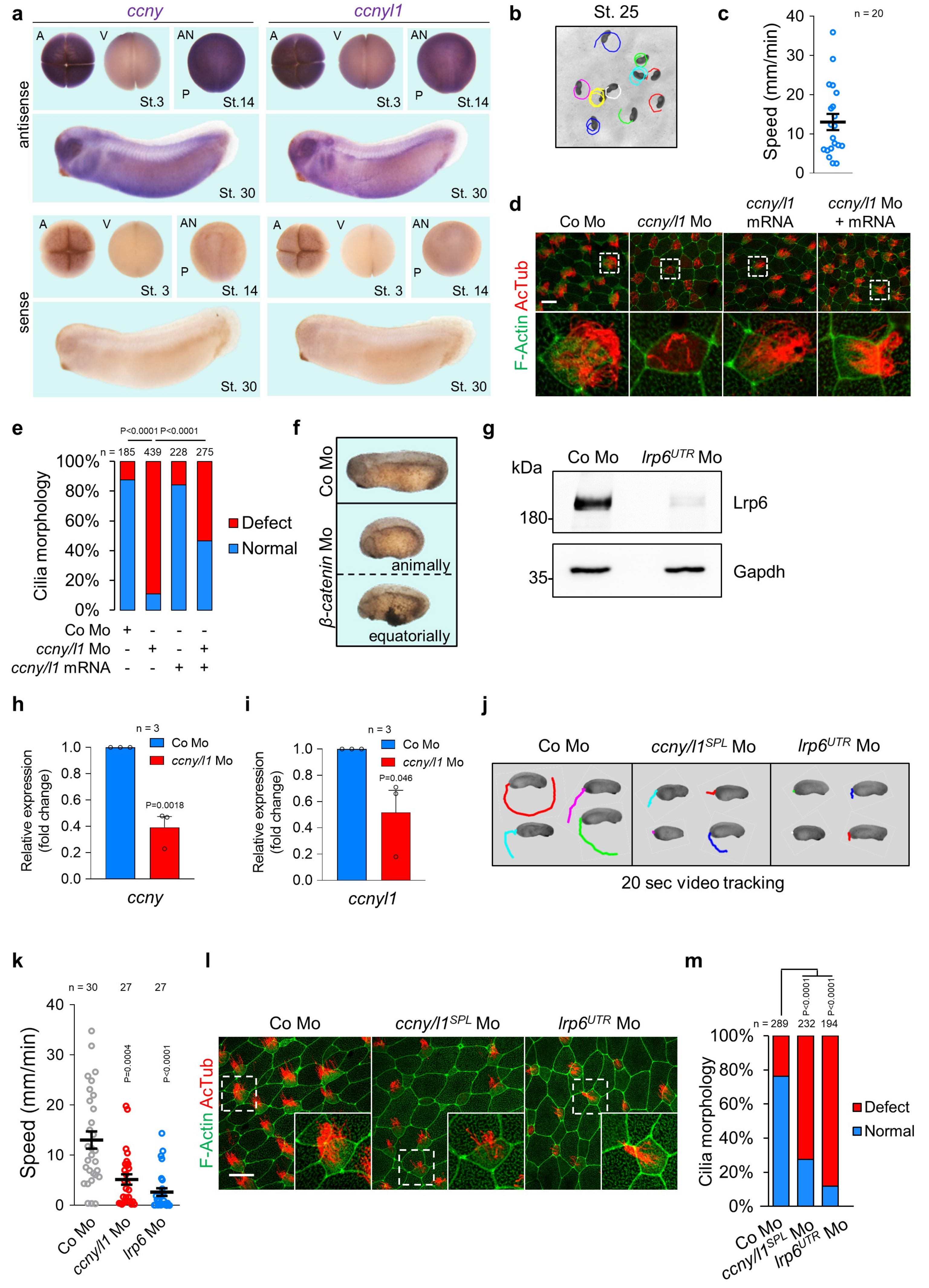

Supplementary Figure 1.

a *Ccny/l1* expression analysis in *X. tropicalis* embryos. WISH of *ccny* and *ccnyl1* in *X. tropicalis* St. 3 (4-cell), St. 14 (neurula) and St. 30 (tadpole) revealing ubiquitous *ccny/l1* expression. A = animal, V = vegetal, AN = anterior.

b-c MCC-driven gliding movements are detectable as early as St. 25. Video-tracking of embryos for 20 seconds. The distance moved is shown by colored line traces and is quantified in (**c**).

d-e Validation of *ccny/l1* Mos. IF for acetylated alpha tubulin (AcTub) and phalloidin (F-actin) on St. 28 *X. tropicalis* MCCs showing rescue of impaired ciliogenesis by *ccny/l1* mRNA co-injection. Scale bar 20 µm. (**e**) Quantification of (**d**). Morphology of cilia classified as normal or defect (shorter and fewer cilia). Two-sided chi square test for statistical analyses. n = number of MCCs analyzed from 3 independent experiments.

f *β-catenin* morphant analysis. *β-catenin* St. 28 morphants show severe axial defects after marginal zone injection. Embryos were injected animally (targeting ectoderm and epidermis) or equatorially (targeting mesoderm).

g-i Validation of *$lrp6^{UTR}$* and *$ccny/l1^{SPL}$* Mos. (**g**) Immunoblot of Lrp6 protein upon *$lrp6^{UTR}$* Mo injection. Gapdh was used as loading control. (**h**) mRNA levels of *ccny* and (**i**) *ccnyl1* in *$ccny/l1^{SPL}$* morphants by RT-qPCR. Two-tailed t-test for statistical analyses.

j-k Independent Mos confirm ciliary gliding defects. Video-tracking of *$ccny/l1^{SPL}$* and *$lrp6^{UTR}$* morphants for 20 seconds. The distance moved is shown by colored line traces and is quantified in (**k**).

l-m Independent Mos confirm cilia morphology defects. IF for AcTub and phalloidin (F-actin) on MCCs of St. 28 *X. tropicalis* embryos showing impaired ciliogenesis in *$ccny/l1^{SPL}$* and *$lrp6^{UTR}$* morphants. Scale bar 20 µm. (**m**) Quantification of (**l**). Morphology of cilia in MCCs classified as normal or defect (shorter and fewer cilia). Two-sided chi square test for statistical analyses. n = number of MCCs analyzed from 3 independent experiments.

Data information: Unless indicated otherwise, unpaired two-tailed t-test for statistical analyses, and data are biological replicates of 3 independent injection series displayed as means ± SEM. Source Data files are available for figures c, e, g, h, i, k and m.

Supplementary Figure 2.

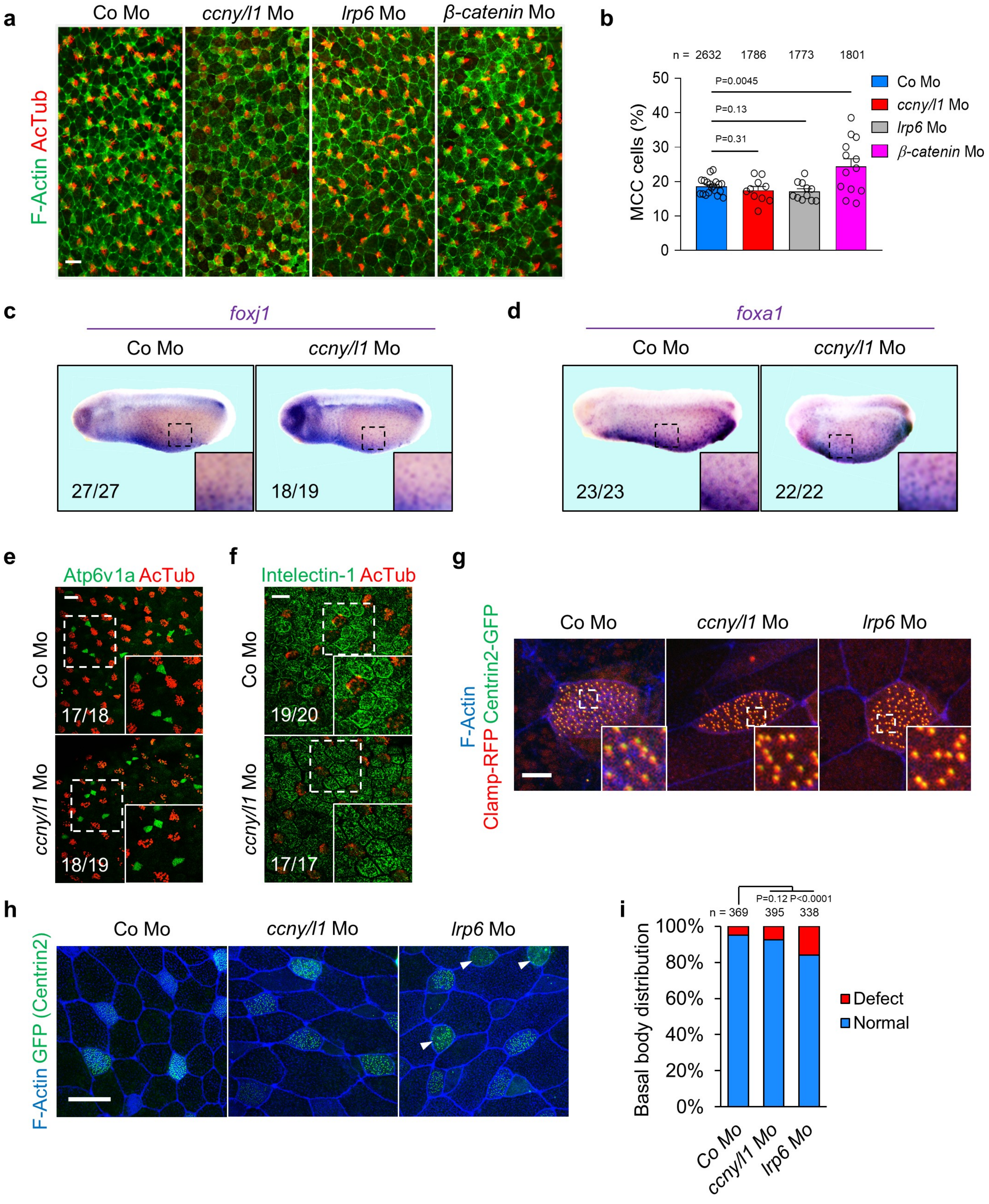

Supplementary Figure 2.

a-c Normal MCC number in *ccny/l1* morphants. IF for AcTub and phalloidin (F-actin) on MCCs from St. 28 *X. tropicalis* embryos (**a**). Scale bar 20 µm. (**b**) Quantification of (**a**). Number of MCCs compared to total epidermal cells. Data are means ± SEM. n = number of total epidermal cells analyzed from 3 independent experiments. Unpaired two-tailed t-test was used for statistical analyses (**c**) Whole-mount *in situ* hybridization of *foxj1* in *ccny/l1* morphants St. 30 tadpoles. Dashed boxes are magnified in insets.

d Normal small secretory cell (SSC) number in *ccny/l1* morphants. Whole-mount *in situ* hybridization of *foxa1* (SSC marker) in *ccny/l1* morphants St. 30 embryos. Dashed boxes are magnified in insets.

e Normal ionocyte (IC) number in *ccny/l1* morphants. IF for Atp6v1a (IC marker) and AcTub in St. 30 *ccny/l1* morphants and control embryos. Scale bar 20 µm. Dashed boxes are magnified in insets. IC number and distribution were quantified in comparison to Co Mo.

f Normal goblet cell number in *ccny/l1* morphants. IF for Intelectin-1 (goblet cell marker) and AcTub in St. 30 *ccny/l1* morphants and control embryos. Scale bar 20 µm. Dashed boxes are magnified in insets. Goblet cell number and distribution were quantified in comparison to Co Mo.

g-i Basal body distribution and polarity in *ccny/l1* and *lrp6* morphants. (**g**) Basal body localization (Centrin2-GFP) and polarity (Clamp-RFP) in MCCs of St. 28 *X. tropicalis* embryos co-injected with Centrin2-GFP, Clamp-RFP and indicated morpholinos. Scale bar 5 µm. (**h**) Centrin2-GFP (basal body marker) co-injected with indicated morpholinos in St. 30 *X. tropicalis* embryos. White arrowheads indicate defective basal body distribution. Scale bar 20 µm. (**i**) Quantification of (**h**). Basal body distribution compared to control embryos. Two-sided chi square test for statistical analyses. n = number of MCCs analyzed from > 15 embryos from 3 independent experiments.

Source Data files are available for figures b, c, d, e, f and i.

Supplementary Figure 3.

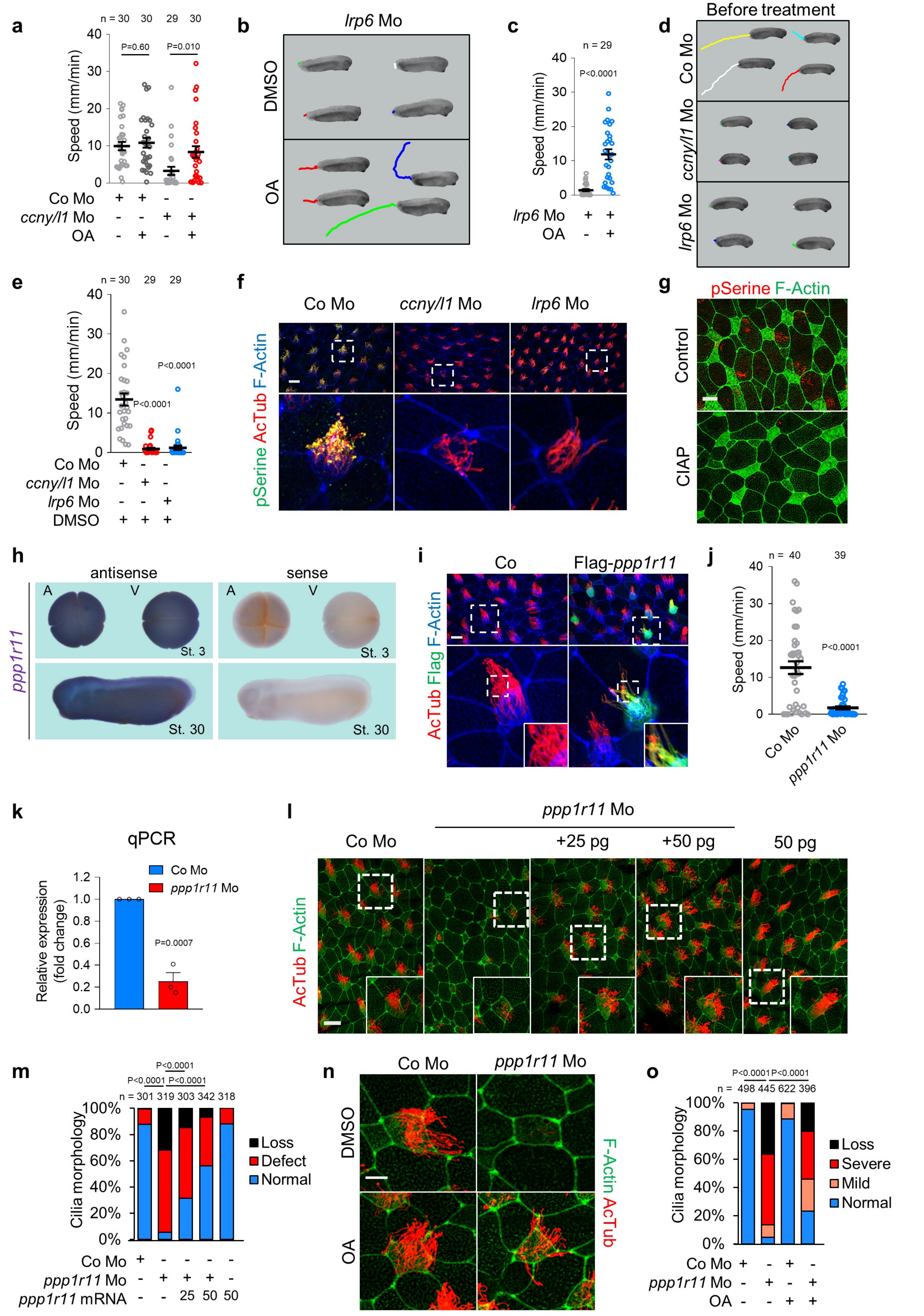

Supplementary Figure 3.

a-e OA rescues movement defects in *ccny/l1* and *lrp6* morphants. (**a**) Quantification of Figure 2b. (**b**) Gliding in St. 32 *lrp6* morphants after treatment with 10 nM OA. (**c**) Quantification of (**b**). (**d-e**) Gliding of St. 26 *ccny/l1* and *lrp6* morphants before treatment with OA**.** Data are displayed as means ± SEM. n = number of embryos analyzed from 3 independent experiments.

f-g Validation of phospho-serine staining. (**f**) Co-IF for pSerine and AcTub. (**g**) IF for pSerine on St. 27 embryos treated with calf intestinal alkaline phosphatase (CIAP). n = 6. Scale bar 20 µm.

h-i Expression analysis of *ppp1r11*. WISH showing *ppp1r11* expression patterns in *X. tropicalis* St. 3 embryos and tadpoles. A = animal, V = vegetal. (**i**) IF for overexpressed Flag-*ppp1r11* in St. 27 MCCs. Scale bar = 20 µm. 8 embryos from 2 independent experiments were analyzed.

j-k Ppp1r11 is required for motile ciliogenesis. Quantification of Figure 2f. Data are displayed as means ± SEM. n = number of embryos analyzed from 4 independent experiments. (**k**) qPCR analysis of *ppp1r11* expression levels after Mo knockdown. Data represented as means ± SEM.

l-m *Ppp1r11* Mo is specific. IF of St. 27 *X. tropicalis* MCCs showing ciliogenesis rescue in *ppp1r11* morphants by human *ppp1r11* mRNA. Scale bar 20 µm. (**m**) Quantification of (**l**). Morphology of cilia classified as normal, defect, or loss. "Defect" and "loss" were grouped for chi square test. n = number of MCCs analyzed from 3 independent experiments.

n-o OA-rescue of cilia in *ppp1r11* morphants. IF of St. 26 MCCs in *ppp1r11* morphants upon OA treatment. Scale bar 5 µm. (**o**) Quantification of (**n**). Morphology of cilia classified as normal, mild, severe or loss. n = number of analyzed MCCs from 3 independent experiments. Two-sided chi square test for statistical analyses, "normal and mild" combined as one group and "severe and loss" as another group.

Data information: Unless indicated otherwise, unpaired two-tailed t-test for statistical analyses. White dashed boxes magnified in lower panels. Source Data files are available for figures a, c, e, j, k, m and o.

Supplementary Figure 4.

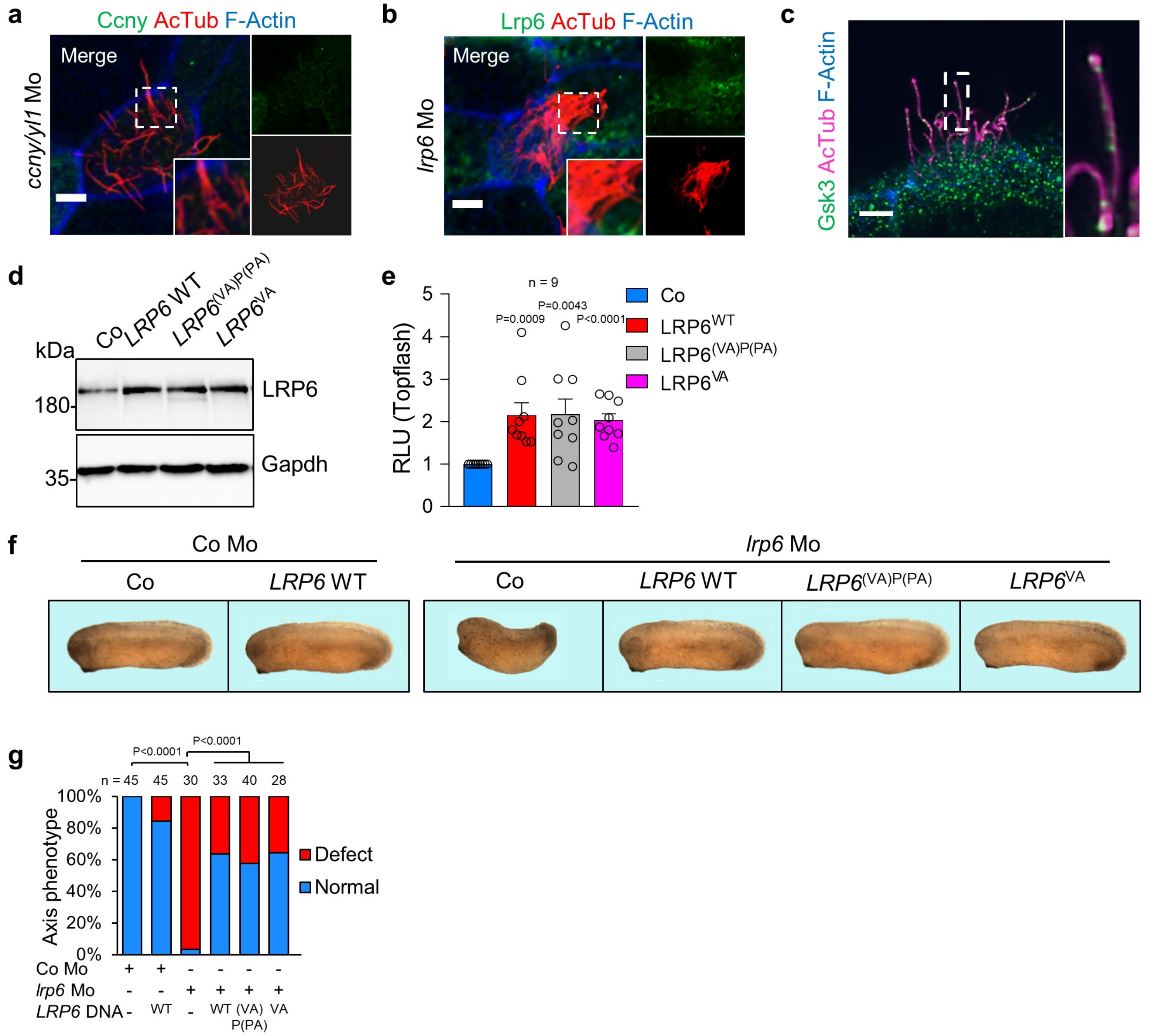

Supplementary Figure 4.

a-b Validation of Ccny and Lrp6 motile cilia staining. Ccny (**a**) and Lrp6 (**b**) were not detected in MCC motile cilia of St. 30 *ccny* or *lrp6* morphants as revealed by IF for acetylated alpha tubulin (AcTub), phalloidin (F-actin) and Ccny or Lrp6, respectively. Scale bar 5 µm. White dashed boxes magnified in lower panels. > 8 embryos were analyzed from 3 independent experiments.

c Gsk3 in motile cilia axonemes. High-resolution IF for Gsk3α/β, AcTub and phalloidin (F-actin) in St. 30 *X. tropicalis* MCCs. Gsk3 was detected at the ciliary axoneme. Scale bar = 5 µm. White dashed boxes magnified in lower panels. 3 independent embryos were analyzed.

d-g LRP6 CTS mutants are expressed and bioactive. (**d**) Immunoblot of LRP6-injected embryos at St. 26 revealed similar protein levels of LRP6 WT, LRP6$^{(VA)P(PA)}$, and LRP6^{VA}. (**e**) Topflash of LRP6-injected embryos showing that CTS mutants activate Wnt signaling. Unpaired two-tailed t-test for statistical analyses. (**f**) *lrp6* St. 30 morphants display axial defects that were rescued with *LRP6* WT, *LRP6*$^{(VA)P(PA)}$ and *LRP6*^{VA} DNA co-injection. (**g**) Quantification of (**f**). Embryos were classified as normal and defect (short, anteriorized body axis). n = number of analyzed embryos. Two-sided chi square test for statistical analyses.

Source Data files are available for figures d, e and g.

Supplementary Figure 5.

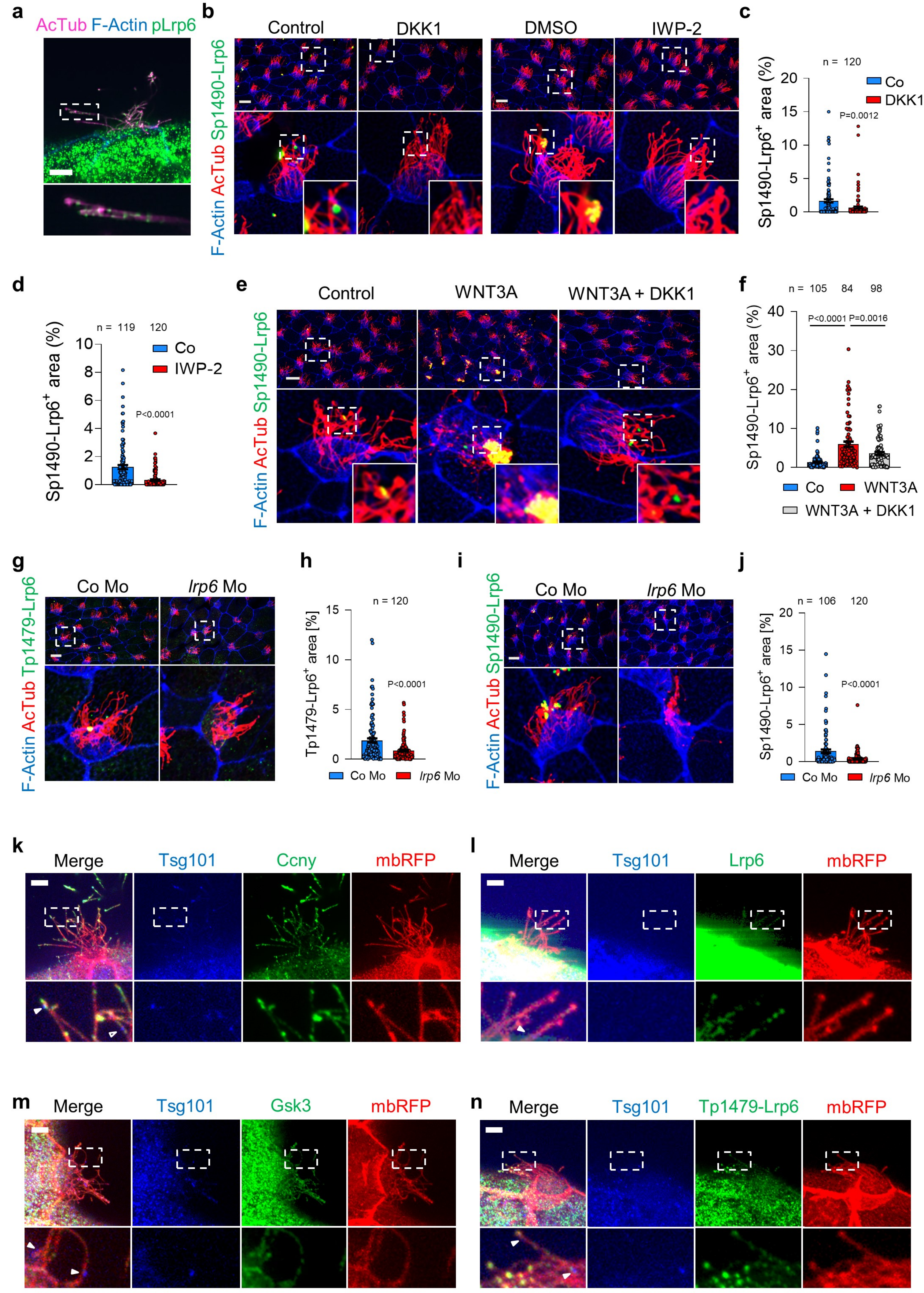

Supplementary Figure 5.

a-f Motile cilia are Wnt-responsive. (**a**) High-resolution IF for Tp1479-Lrp6 in St. 30 *X. tropicalis* epidermis. Scale bar = 5 µm. Dashed box magnified in inset. (**b**) IF for Sp1490-Lrp6 in St. 30 *X. tropicalis* MCCs following 2 hour treatments with DKK1 and IWP-2. Scale bar = 20 µm. (**c-d**) Quantification of (**b**). Sp1490-Lrp6 staining in embryos treated with DKK1 (**c**) and IWP-2 (**d**). Data show the staining area of Sp1490-Lrp6 relative to the AcTub area for individual MCCs and are displayed as means ± SEM. n = number of MCCs analyzed from 3 independent experiments. (**e-f**) IF for Sp1490-Lrp6 in St. 30 *X. tropicalis* MCCs following 2 hour treatment with WNT3A ± DKK1. Scale bar = 20 µm. (**f**) Quantification of (**e**). Data shows the staining area of Sp1490-Lrp6 relative to the AcTub area for individual MCCs, displayed as means ± SEM. n = number of MCCs analyzed from 3 independent experiments.

g-j Validation of Tp1479-Lrp6 and Sp1490-Lrp6 motile cilia staining. (**g**) Tp1479-Lrp6 was reduced in MCC motile cilia of St. 30 *ccny* morphants as revealed by IF for Tp1479-Lrp6. Scale bar 5 µm. (**h**) Quantification of (**g**). Data shows the staining areas of Tp1479-Lrp6 relative to AcTub for individual MCCs, displayed as means ± SEM. n = number of MCCs analyzed from 3 independent experiments. (**i**) Sp1490-Lrp6 was reduced in MCC motile cilia of St. 30 *lrp6* morphants as revealed by Sp1490-Lrp6 staining. Scale bar 5 µm. (**j**) Quantification of (**i**). Data shows the staining areas of Sp1490-Lrp6 relative to AcTub for individual MCCs, displayed as means ± SEM. n = number of MCCs analyzed from 3 independent experiments.

k-n Wnt components do not co-localize with exovesicles. Co-IF for Tsg101 (exovesicle marker) with Ccny, Lrp6, Tp1479-Lrp6 and Gsk3 in St. 30 *X. tropicalis* epidermis after mbRFP injections. White arrowheads point to exovesicles. Scale bar = 5 µm. 3 independent embryos were analyzed.

Data information : Unpaired two-tailed t-test for all statistical analyses. White dashed boxes magnified in lower panels. Source Data files are available for figures c, d, f, h and j.

Supplementary Figure 6.

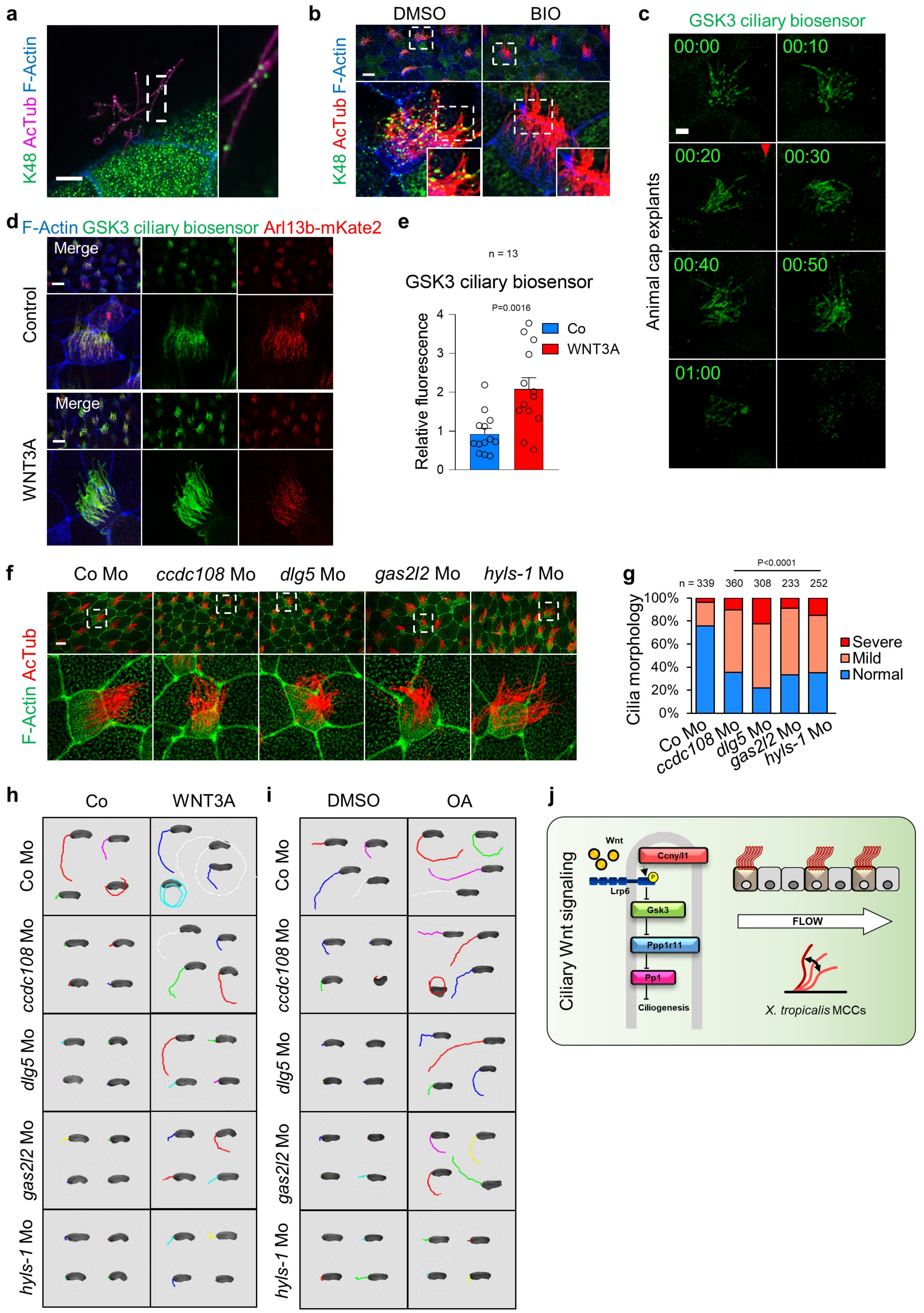

Supplementary Figure 6.

a-b Wnt signaling reduces protein ubiquitination in motile cilia. (**a**) High-resolution IF for K48 ubiquitin in St. 30 *X. tropicalis* MCCs. Scale bar = 5 µm. 3 embryos were analyzed. (**b**) K48 staining after BIO treatment. Scale bar = 20 µm. White dashed boxes magnified in lower panels. 9 embryos from 3 independent experiments were analyzed.

c GSK3 ciliary biosensor Co treatment. Still images of GSK3 ciliary biosensor fluorescence acquired during live cell imaging of motile cilia in animal cap explants at St. 30 equivalent. Control buffer was added after 27 min of imaging. GFP flourescence eventually decays due to photobleaching.

d-e GSK3 ciliary biosensor in fixed embryos confirms Wnt induction. IF of fixed St. 30 embryos injected with GSK3 ciliary biosensor and pArl13b-mKate2 after 30 min treatment with WNT3A recombinant protein or control. Scale bar = 20 µm. (**e**) Quantification of (**d**). Data show the mean relative fluorescence of the biosensor normalized to mean Arl13b-mKate2 signal intensity per image. n = number of embryos from 4 independent experiments. Unpaired two-tailed t-test for statistical analyses.

f-g Doses of ciliopathy-related morpholinos employed cause modest cilia abnormalities. IF of *X. tropicalis* epidermis injected as indicated and stained with AcTub and phalloidin for F-actin at St. 30. (**g**) Quantification of (**f**). Morphology of cilia in MCCs was classified as normal, mild (> half the length compared to control) or severe (< half the length compared to control). n = number of analyzed MCCs from > 10 embryos from 3 independent experiments. Two-sided chi square test for statistical analyses, "mild and severe" combined as one group vs. "normal".

h-i WNT3A reverses ciliopathy- and fertility- related mucociliary gliding defects. Gliding of ciliopathy morphants after 1h treatment with WNT3A (**h**) or OA (**i**). Embryos were video-tracked for 20 sec at St. 28. The distance moved by 4 representative embryos each is shown by colored line traces.

j Ciliary Wnt signaling is required for ciliogenesis and cilia movements. Schematic of ciliary Wnt signaling. Wnt promotes cilia beating and ciliogenesis.

Source Data files are available for figures e and g.
