# Supplementary material for: Mucociliary Wnt signaling promotes cilia biogenesis and beating
Source: Nat Commun. 2023 Mar 6;14:1259. doi: 10.1038/s41467-023-36743-2 (PMC9988884; doi:10.1038/s41467-023-36743-2)
Supplement: Supplementary file 2 — Description of Additional Supplementary Files [file 41467_2023_36743_MOESM2_ESM.pdf]

File name: Supplementary Movie 1

Description: related to Figure 1. High speed time lapse sequences of *X. tropicalis* MCCs injected with control (Co) morpholino (Mo). Video was recorded with 993 frames per second (FPS). One second (993 frames) is shown in slow motion of 60 FPS.

File name: Supplementary Movie 2

Description: related to Figure 1. High speed time lapse sequences of *X. tropicalis* MCCs injected with *ccny/l1* Mo. Video was recorded with 993 frames per second (FPS). One second (993 frames) is shown in slow motion of 60 FPS.

File name: Supplementary Movie 3

Description: related to Figure 1. High speed time lapse sequences of *X. tropicalis* MCCs injected with *lrp6* Mo. Video was recorded with 993 frames per second (FPS). One second (993 frames) is shown in slow motion of 60 FPS.

File name: Supplementary Movie 4

Description: related to Figure 1. High speed time lapse sequences of *X. tropicalis* MCCs injected with *β-catenin* Mo. Video was recorded with 993 frames per second (FPS). One second (993 frames) is shown in slow motion of 60 FPS.

File name: Supplementary Movie 5

Description: related to Figure 2. Movement tracking video of *X. tropicalis* embryos injected with Co Mo and treated with DMSO.

File name: Supplementary Movie 6

Description: related to Figure 2. Movement tracking video of *X. tropicalis* embryos injected with *ccny/l1* Mo and treated with DMSO.

File name: Supplementary Movie 7

Description: related to Figure 2. Movement tracking video of *X. tropicalis* embryos injected with *lrp6* Mo and treated with DMSO.

File name: Supplementary Movie 8

Description: related to Figure 2. Movement tracking video of *X. tropicalis* embryos injected with *ccny/l1* Mo and treated with Okadaic acid (OA). OA treatment rescues movement defects in *ccny/l1* morphants.

File name: Supplementary Movie 9

Description: related to Figure 2. Movement tracking video of *X. tropicalis* embryos injected with *lrp6* Mo and treated with Okadaic acid (OA). OA treatment rescues movement defects in *lrp6* morphants.

File name: Supplementary Movie 10

Description: related to Figure 4. Live cell imaging of motile cilia in animal cap explants from *X. tropicalis* embryos injected with GSK3 ciliary (GFP-) biosensor (green). Recombinant WNT3A added at 30 mins. Shown is one representative multiciliated cell. Each frame = 5 mins. Note increase in GSK3 (GFP-) biosensor signal upon WNT3A addition. The decrease in signal after 1:15 h is due to photobleaching, as observed in non-WNT3A treated controls (Supplementary Figure 6c).

File name: Supplementary Movie 11

Description: related to Figure 4. Movement tracking videos of *X. tropicalis* wild-type embryos treated with control buffer (Co). Embryos were tracked for 20 seconds.

File name: Supplementary Movie 12

Description: related to Figure 4. Movement tracking videos of *X. tropicalis* wild-type embryos treated with WNT3A recombinant protein. Accelerated gliding is observed after 2h treatment with WNT3A recombinant protein. Embryos were tracked for 20 seconds.

File name: Supplementary Movie 13

Description: related to Figure 4. Movement tracking videos of *X. tropicalis* wild-type embryos treated with WNT3A + DKK1. Accelerated gliding effect after 2h treatment with WNT3A recombinant protein was reversed by co-treatment with DKK1. Embryos were tracked for 20 seconds.

File name: Supplementary Movie 14

Description: related to Figure 4. High speed time lapse sequence of *X. tropicalis* MCCs treated with control buffer (Co). Video was recorded with overall 993 frames per second (FPS). One second (993 frames) is shown in slow motion of 60 FPS.

File name: Supplementary Movie 15

Description: related to Figure 4. High speed time lapse sequence of *X. tropicalis* MCCs treated with WNT3A recombinant protein. Video was recorded with overall 993 frames per second (FPS). One second (993 frames) is shown in slow motion of 60 FPS.

File name: Supplementary Movie 16

Description: related to Figure 4. High speed time lapse sequence of *X. tropicalis* MCCs treated with WNT3A + DKK1. Video was recorded with overall 993 frames per second (FPS). One second (993 frames) is shown in slow motion of 60 FPS.

File name: Supplementary Movie 17

Description: related to Figure 4. Representative movie of human respiratory epithelia culture after 2h treatment with control buffer (Co). 2.6 seconds with 200 FPS are shown.

File name: Supplementary Movie 18

Description: related to Figure 4. Representative movie of human respiratory epithelia culture after 2h treatment with WNT3A recombinant protein. 2.6 seconds with 200 FPS are shown.
