# Supplementary material for: Mucociliary Wnt signaling promotes cilia biogenesis and beating
Source: Nat Commun. 2023 Mar 6;14:1259. doi: 10.1038/s41467-023-36743-2 (PMC9988884; doi:10.1038/s41467-023-36743-2)
Supplement: Supplementary file 21 — Reporting Summary [file 41467_2023_36743_MOESM21_ESM.pdf]

nature portfolio

Corresponding author(s): Christof Niehrs

Last updated by author(s): Feb 6, 2023

Reporting Summary

Nature Portfolio wishes to improve the reproducibility of the work that we publish. This form provides structure for consistency and transparency in reporting. For further information on Nature Portfolio policies, see our Editorial Policies and the Editorial Policy Checklist.

Statistics

For all statistical analyses, confirm that the following items are present in the figure legend, table legend, main text, or Methods section.

n/a	Confirmed	
☐	☒	The exact sample size (*n*) for each experimental group/condition, given as a discrete number and unit of measurement
☐	☒	A statement on whether measurements were taken from distinct samples or whether the same sample was measured repeatedly
☐	☒	The statistical test(s) used AND whether they are one- or two-sided<br>*Only common tests should be described solely by name; describe more complex techniques in the Methods section.*
☒	☐	A description of all covariates tested
☐	☒	A description of any assumptions or corrections, such as tests of normality and adjustment for multiple comparisons
☐	☒	A full description of the statistical parameters including central tendency (e.g. means) or other basic estimates (e.g. regression coefficient) AND variation (e.g. standard deviation) or associated estimates of uncertainty (e.g. confidence intervals)
☐	☒	For null hypothesis testing, the test statistic (e.g. *F*, *t*, *r*) with confidence intervals, effect sizes, degrees of freedom and *P* value noted<br>*Give P values as exact values whenever suitable.*
☒	☐	For Bayesian analysis, information on the choice of priors and Markov chain Monte Carlo settings
☒	☐	For hierarchical and complex designs, identification of the appropriate level for tests and full reporting of outcomes
☐	☒	Estimates of effect sizes (e.g. Cohen's *d*, Pearson's *r*), indicating how they were calculated

Our web collection on statistics for biologists contains articles on many of the points above.

Software and code

Policy information about availability of computer code

Data collection	AxioVision 40 version 4.8.2.0 was used to collect data for ciliary gliding assay, for imaging phenotypes and in situ hybridizations.<br>Zeiss ZEN 2012 (black edition) version 2.5 was used for confocal imaging on LSM700 and LSM780.<br>Image Reader LAS-300 version 2.21 was used to acquire western blot images.<br>Nis Elements AR version 5.02 was used for high-resolution confocal imaging and high-speed video microscopy of Xenopus motile cilia.<br>SAVA software version 2.08W was used for imaging of human airway motile cilia.<br>Zeiss Leo 1530 SEM software control tool was used to generate SEM images.<br>Spark Control version 2.3 was used to measure luciferase activity in Topflash assays.<br>Roche Light Cycler 480 Software version 1.5.1 was used to generate real-time quantitative PCR data.
Data analysis	GraphPad Prism 7 software version 7.03 was used to generate graphs and statistics.<br>FIJI (ImageJ) software version 1.53i was used for confocal imaging analysis and tracking of embryos in gliding assays as well as western blot analysis.<br>SAVA software version 2.0.8W was used for CBF analysis in human airway epithelia and Xenopus MCCs.<br>Adobe Photoshop CS6 software version 13.0 x64 was used to edit the background in representative WISH, phenotype and gliding assay embryos.<br>Zeiss ZEN 2012 (black edition) version 2.5 was used to analyze live cell imaging and confocal imaging.<br>Nis-Elements AR version 5.02 was used to deconvolute and analyze high-resolution confocal images and high-speed video microscopy of Xenopus MCCs.<br>Zeiss Leo SEM software control version 1530 was used to analyze SEM images.

For manuscripts utilizing custom algorithms or software that are central to the research but not yet described in published literature, software must be made available to editors and reviewers. We strongly encourage code deposition in a community repository (e.g. GitHub). See the Nature Portfolio guidelines for submitting code & software for further information.

Data

Policy information about availability of data

All manuscripts must include a data availability statement. This statement should provide the following information, where applicable:

- Accession codes, unique identifiers, or web links for publicly available datasets
- A description of any restrictions on data availability
- For clinical datasets or third party data, please ensure that the statement adheres to our policy

NCBI Reference Sequences for cloning:
Human lrp6: https://www.ncbi.nlm.nih.gov/nuccore/BC143725.1
Xenopus tropicalis ppp1r11: https://www.ncbi.nlm.nih.gov/nuccore/NM_001004815.1
Human ppp1r11: https://www.ncbi.nlm.nih.gov/nuccore/NM_021959.3

Databases for information search:
https://www.xenbase.org/entry/

No third party datasets were analyzed in this study.

All original data and raw microscopy images are available from the corresponding author upon request. Source data for all graphs and blot are provided with this paper in the Source Data file.

Human research participants

Policy information about studies involving human research participants and Sex and Gender in Research.

Reporting on sex and gender	Sex and gender cannot be determined in Xenopus embryonic stages used in this study.
Population characteristics	n/a
Recruitment	n/a
Ethics oversight	n/a

Note that full information on the approval of the study protocol must also be provided in the manuscript.

Field-specific reporting

Please select the one below that is the best fit for your research. If you are not sure, read the appropriate sections before making your selection.

☒ Life sciences ☐ Behavioural & social sciences ☐ Ecological, evolutionary & environmental sciences

For a reference copy of the document with all sections, see nature.com/documents/nr-reporting-summary-flat.pdf

Life sciences study design

All studies must disclose on these points even when the disclosure is negative.

Sample size

No sample-size calculation was performed due to the nature of this study. Sample size was following Xenopus literature standards, and it was sufficient to execute reliable statistical analyses:
For qualitative (high-resolution) confocal imaging minimum 3 independent embryos were analyzed.
For quantitative confocal imaging MCCs of 9 or more embryos from 3 independent injections were analyzed.
For gliding assays minimum 20 embryos from 3 independent injections were analyzed.
For whole-mount in situ hybridization minimum 19 embryos from 3 independent injections were analyzed.
For phenotypical analyses minimum 20 embryos from 2-3 independent injections were analyzed.
Human airway epithelial MCC analysis was performed in 3-9 samples from 2 different donors.

Independent injections mean that the experiments were performed on different days with different male and female frogs.

Data exclusions

In all ciliary gliding assays, embryos that woke up from anesthesia were excluded because first muscle-swimming movements would interfere with the analysis of cilia-based embryo gliding. Significant outliers were identified and eliminated by Grubb's test to avoid extreme values that are caused by e.g. sticky surface, uneven agar plate, waking up from anesthesia.

Replication

Ciliary movement assays were repeated 3-4 times independently. All assays for immunofluorescent microscopy were repeated 3-5 times independently. SEM imaging was repeated 2 times independently. All western blot analyses (incl. IP of Flag-Ppp1r11) were repeated 3-4 times independently. Live cell imaging of GSK3 ciliary biosensor was repeated 3 times independently. Xenopus in situ hybridization was repeated 2-3 times independently. Xenopus phenotype analyses were repeated 2-3 times independently. Xenopus qRT-PCR was performed 3 times

independently. Topflash of Xenopus embryos was performed 3 times independently. High-speed video microscopy was repeated 3 times independently. CBF measurements in human respiratory epithelia ALI cultures were repeated 3-9 times from 2 different donors. We confirm that the above mentioned experiments showed similar results with the same conclusion. Replicate numbers for each panel are indicated in the figures and corresponding legends.

Randomization

We split cultured cells and fertilized Xenopus eggs equally and randomly allocated them to each experimental group.

Blinding

Scoring of Xenopus phenotypes, WISH and IF image analyses were performed under blinding. All folders of IF images were duplicated and renamed by a person that was not involved in this study before quantifications. Phenotypes and WISH embryos were renamed group-wise in cell culture dishes by a person that was not invovled in this study. During data collection, blinding was not possible due to the nature of the experiments.

Reporting for specific materials, systems and methods

We require information from authors about some types of materials, experimental systems and methods used in many studies. Here, indicate whether each material, system or method listed is relevant to your study. If you are not sure if a list item applies to your research, read the appropriate section before selecting a response.

Materials & experimental systems

n/a	Involved in the study
☐	☒ Antibodies
☐	☒ Eukaryotic cell lines
☒	☐ Palaeontology and archaeology
☐	☒ Animals and other organisms
☒	☐ Clinical data
☒	☐ Dual use research of concern

Methods

n/a	Involved in the study
☒	☐ ChIP-seq
☒	☐ Flow cytometry
☒	☐ MRI-based neuroimaging

Antibodies

Antibodies used

1. Mouse monoclonal anti-acetylated alpha tubulin; clone 6-11B-1; Sigma-Aldrich; Cat#T7451
2. Rabbit polyclonal anti-LRP6; clone C5C7; Cell Signaling Technology; Cat#2560; Lot 11
3. Rabbit monoclonal anti-GAPDH; clone 14C10; Cell Signaling Technology; Cat#2118S; Lot: 10
4. Rabbit polyclonal anti-CCNY; homemade; Davidson et al., 2009
5. Mouse monoclonal anti-GSK3β; clone 7/GSK-3b; BD Biosciences; Cat# 610201; Lot: 6125651
6. Rabbit monoclonal anti-GSK3α/β; clone D75D3; Cell Signaling Technology; Cat# 5676; Lot: 6
7. Rabbit polyclonal anti-Phospho-LRP6 (S1490); Cell Signaling Technology; Cat#2568S; Lot: 6
8. Rabbit polyclonal anti-Phospho-LRP6 (T1479); Homemade; Davidson et al., 2005
9. Mouse monoclonal anti-FLAG; clone M2; Sigma Aldrich; Cat#F3165; Lot: SLBN8915V
10. Mouse monoclonal anti-Phosphoserine; clone 4A4; Millipore; Cat#05-1000; Lot: 3280139
11. Mouse monoclonal anti-Phosphothreonine; clone Q7; Qiagen; Cat#37420; Lot: 11546905
12. Rabbit polyclonal anti-ATP6V1A; Abcam; Cat#ab137574; Lot: GR220966-33
13. Rabbit monoclonal anti-Ubiquitin, Lys48-Specific; clone Apu2; Millipore; Cat#05-1307; Lot: 2652959
14. Rabbit polyclonal Anti-ITLN1; Proteintech; Cat#11770-1-AP; Lot: Lot: 00017014
15. Rabbit monoclonal anti-Acetyl-α-Tubulin (Lys40); clone D20G3; Cell Signaling Technology; Cat#5335T; Lot: 6
16. Mouse monoclonal anti-TSG101; clone 4A10; Abcam; Cat#ab83; Lot: GR32227-2
17. Phalloidin-iFluor 488 Reagent; Abcam; Cat#ab176753; Lot: GR3324781-1
18. Phalloidin-iFluor 405 Reagent; Abcam; Cat#ab176752; Lot: GR3249833-16
19. Goat anti-mouse IgG (H+L) HRP; Jackson ImmunoResearch; Cat#115-035-146
20. Goat anti-rabbit IgG (H+L) HRP; Jackson ImmunoResearch; Cat#111-035-144
21. Donkey anti-Goat IgG-HRP; Santa Cruz; Cat#sc-2020
22. Donkey polyclonal Anti-Mouse IgG, Alexa Fluor 647; Jackson ImmunoResearch; Cat#715-605-151
23. Goat polyclonal Anti-Rabbit IgG, Alexa Fluor 488; Invitrogen; Cat#A11008
24. Anti-Digoxigenin-AP, Fab-Fragments; Roche; Cat#11093274910; Lot: 16646820

Validation

Validations are described for all antibodies in the same order as indicated above:

1. Tested by manufacturer in Western Blotting and Immunofluorescence using HeLa, U87, COS7, P19, RAT2, CHO, MDBK, MDCK; Confirmed reactivitiy in bovine, frog, invertebrates, human, hamster, mouse, protista, pig, monkey, chicken, rat, plant.
2. Tested by manufacturer in HepG2, HeLa, Rat2 using western blotting. We validated in Xenopus by Morpholino KD.
3. Tested by manufactures in HeLa, NIH/3T3, C6, HUVEC, L929 cells using western blotting. Commonly used and cited more than 2000 times.
4. Homemade antibody validated in Xenopus, Drosophila cells and human cell lines (HEK 293, HeLa, P19) in Davidson et al., 2009 using western blotting. We validated by Morpholino KD.
5. Tested by manufacturer in HeLa (IF staining) and RSV-3T3 lysate (western blotting). We validated in Figure 2i.
6. Tested by manufactures in HeLa, NIH/3T3, C6, CHO, COS-7 cells using western blotting. Commonly used antibody, cited more than 200 times.
7. Tested by manufacturer in HeLa cells using western blotting and in Xenopus lysate western blotting by Davidson et al. 2009.

Commonly used and cited more than 160 times.
8. Homemade antibody validated in human cell lines (HEK 293, HeLa, P19) in Davidson et al., 2005 and Davidson et al., 2009 by western blotting.
9. Commonly used and cited more than 3000 times. Validated by the manufacturer in immunofluorescence in canine kidney epithelial cells. Described by the manufacturer as reactive to all species. We validated by IF and comparison to control (without Flag injection).
10. Tested by the manufacturer detecting 10 active kinases in denatured western blot conditions and by immunofluerescence in 293 cells.
11. Tested by manufacturer in COS-7, NIH, 293, Huh-7, HeLa S3, HeLa Acc, COS lysates using western blotting.
12. Tested by manufacturer in HeLa and HepG2 cells by western blotting and IF.
13. Tested by manufacturer in NIH/3T3, HeLa and A431 cells by IF. We confirmed in Xenopus by BIO treatment.
14. Validated in Xenopus epidermis immunofluorescent staining by Kim et al., Nature Communications, 2020.
15. Tested by manufacturer in human cell lines and mouse testis by IF and western blotting. Commonly used and cited more than 28 times.
16. Tested by manufacturer in human cell lines and mouse by western blotting. Commonly used and cited more than 210 times.
17. Tested by manufacturer in macrophage J774A.1 and HeLa cells by IF. Commonly used and cited more than 120 times.
18. Tested by manufacturer in Bovine Foetal Aeorta Endothelial (BFA) cells by IF. Commonly used and cited more than 10 times.
19. Commonly used and cited more than 400 times. Validated by western blotting in human cell lines on the manufacturers homepage.
20. Commonly used and cited more than 1600 times. Validated by western blotting in human cell lines on the manufacturers homepage.
21. Tested by manufacturer in A-431, HeLa and Sol8 whole cell lysates by western blotting.
22. Commonly used and cited more than 130 times. The antibody has been tested by the manufacturer in ELISA and they ensure minimal cross-reaction with bovine, chicken, goat, guinea pig, syrian hamster, horse, human, rabbit, rat and sheep serum proteins.
23. Commonly used and cited more than 6000 times. Confirmed by the manufacturer in human cell lines and mouse/rat cryosections by IF.
24. The antibody was been first validated for WISH in the Atlantic halibut by Eilertsen M, et al. 2014 and in Danio rerio by Carlisle et al., 2013. It is commonly used for WISH in Xenopus embryos (see e.g. Lee et al., Nat. Comm. 2020).

Eukaryotic cell lines

Policy information about cell lines and Sex and Gender in Research

Cell line source(s)	HEK293T (ATCC); Primary human respiratory epithelial cells from healthy donors (UKM Münster)
Authentication	None of the cell lines were authenticated.
Mycoplasma contamination	All the cells were tested regularly and negative for mycoplasma contamination.
Commonly misidentified lines (See ICLAC register)	No commonly misidentified lines used in this study.

Animals and other research organisms

Policy information about studies involving animals; ARRIVE guidelines recommended for reporting animal research, and Sex and Gender in Research

Laboratory animals	Xenopus tropicalis frogs were obtained from Nasco, the National Xenopus Resource (NXR) and the European Xenopus Resource Centre (EXRC). Adults female Xenopus tropicalis were used to obtain eggs. Adults male Xenopus tropicalis were used to obtain testis for in vitro fertilization. All animal experiments were approved by the state review board of Baden-Württemberg, Regierungspräsidium Karlsruhe, Germany (permit number 35-9185.81/G-141/18).
Wild animals	The study did not involve wild animals.
Reporting on sex	Sex of the Xenopus embryos is not distinguishable at these embryonic stages and irrelevant to the study.
Field-collected samples	The study did not involve field-collected samples.
Ethics oversight	The state review board of Baden-Wurttemberg, Germany (Permit number 35-9185.81/G-141/18 (Regierungspräsidium Karlsruhe))

Note that full information on the approval of the study protocol must also be provided in the manuscript.
